# Supplementary material for: Kir4.1-Dependent Astrocyte-Fast Motor Neuron Interactions Are Required for Peak Strength
Source: Neuron. 2018 Apr 18;98(2):306–319.e7. doi: 10.1016/j.neuron.2018.03.010 (PMC5919779; doi:10.1016/j.neuron.2018.03.010)
Supplement: Document S1. Figures S1–S7 and Tables S1 and S2 [file mmc1.pdf]

Neuron, Volume *98*

Supplemental Information

Kir4.1-Dependent Astrocyte-Fast Motor Neuron Interactions Are Required for Peak Strength

Kevin W. Kelley, Lucile Ben Haim, Lucas Schirmer, Giulia E. Tyzack, Michaela Tolman, John G. Miller, Hui-Hsin Tsai, Sandra M. Chang, Anna V. Molofsky, Yongjie Yang, Rickie Patani, Andras Lakatos, Erik M. Ullian, and David H. Rowitch

Supplemental figures

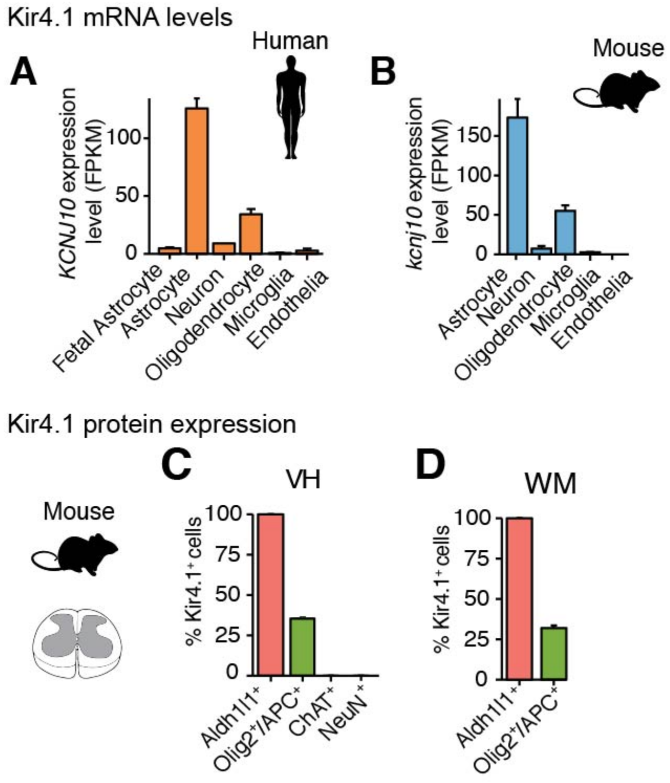

Figure S1 | *Kir4.1* is predominantly expressed in AS. Related to Figure 1. A, B) *Kir4.1* mRNA is highly expressed in cortical (CTX) AS compared to other major CNS cell types both in humans (**A**) (Zhang et al., 2016) and mice (Zhang et al., 2014) (**B**) (mean ± sem). **C, D)** Histological counts of Kir4.1 percent positive AS (Aldh1l1$^+$), oligodendrocytes (Olig2$^+$/APC (CC1)$^+$), neurons (NeuN$^+$) and MN (ChAT$^+$) in ventral horn (VH) (**C**) and white matter (WM) (**D**) of lumbar mouse spinal cord at P16 for AS counts and P30 for oligodendrocyte and neuron counts (n = 3 mice, mean ± sem).

Kir4.1 developmental expression pattern in relation to VGLUT1

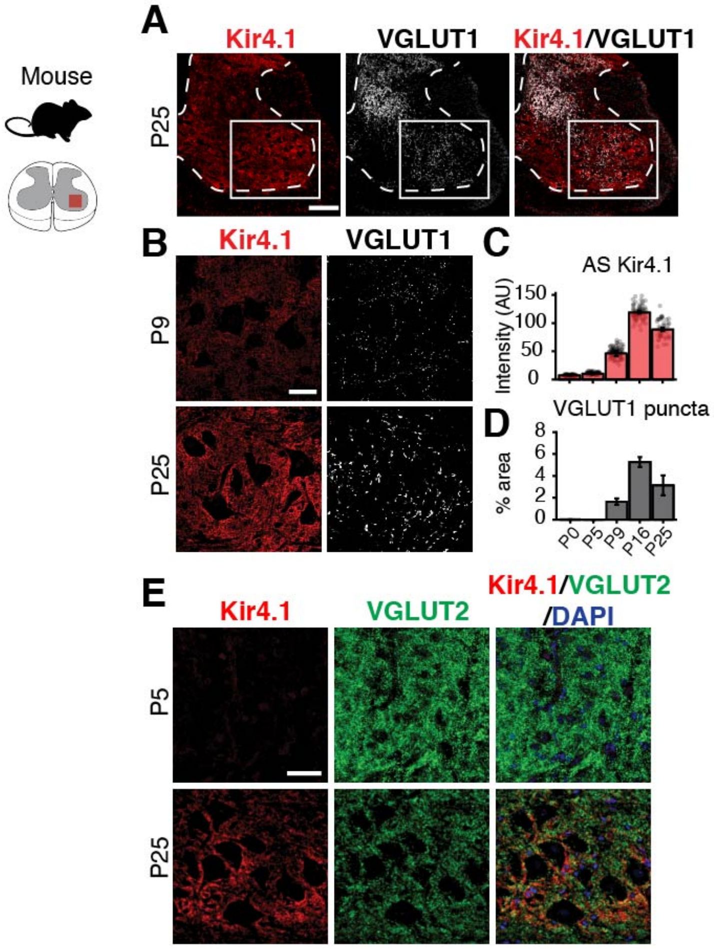

Figure S2 | Postnatal expression of AS Kir4.1 in relation to VGLUT1 and VGLUT2. Related to Figure 1. A) Immunofluorescence staining of Kir4.1 and VGLUT1 in the mouse spinal cord at P25 showing enrichment of both proteins in the ventral horn (scale bar: 200µm). **B**) Synchronized developmental upregulation of Kir4.1 and VGLUT1 in ventral lumbar spinal cord between P9 and P25 (scale bar: 40µm). **C, D)** Quantification of Kir4.1 immunofluorescence intensity per AS (*Aldh1l1-GFP*$^{+}$) (**C**) and the fraction of area occupied by VGLUT1 puncta (**D**) at the indicated developmental ages (n = 3 animals, > 50 AS counts/animal, mean ± sem). **E**) Developmental upregulation of Kir4.1 in the ventral horn does not correlate with VGLUT2 puncta expression.

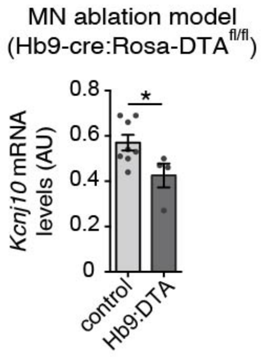

Figure S3 | MNs are necessary for *Kir4.1* expression in the ventral spinal cord. Related to Figure 1. *Kcnj10* mRNA levels are strongly downregulated in mice lacking MNs (Hb9:cre:Rosa-DTA $^{fl/fl}$) at E18.5 (n=4-8, mean ± sem, Student t-test).

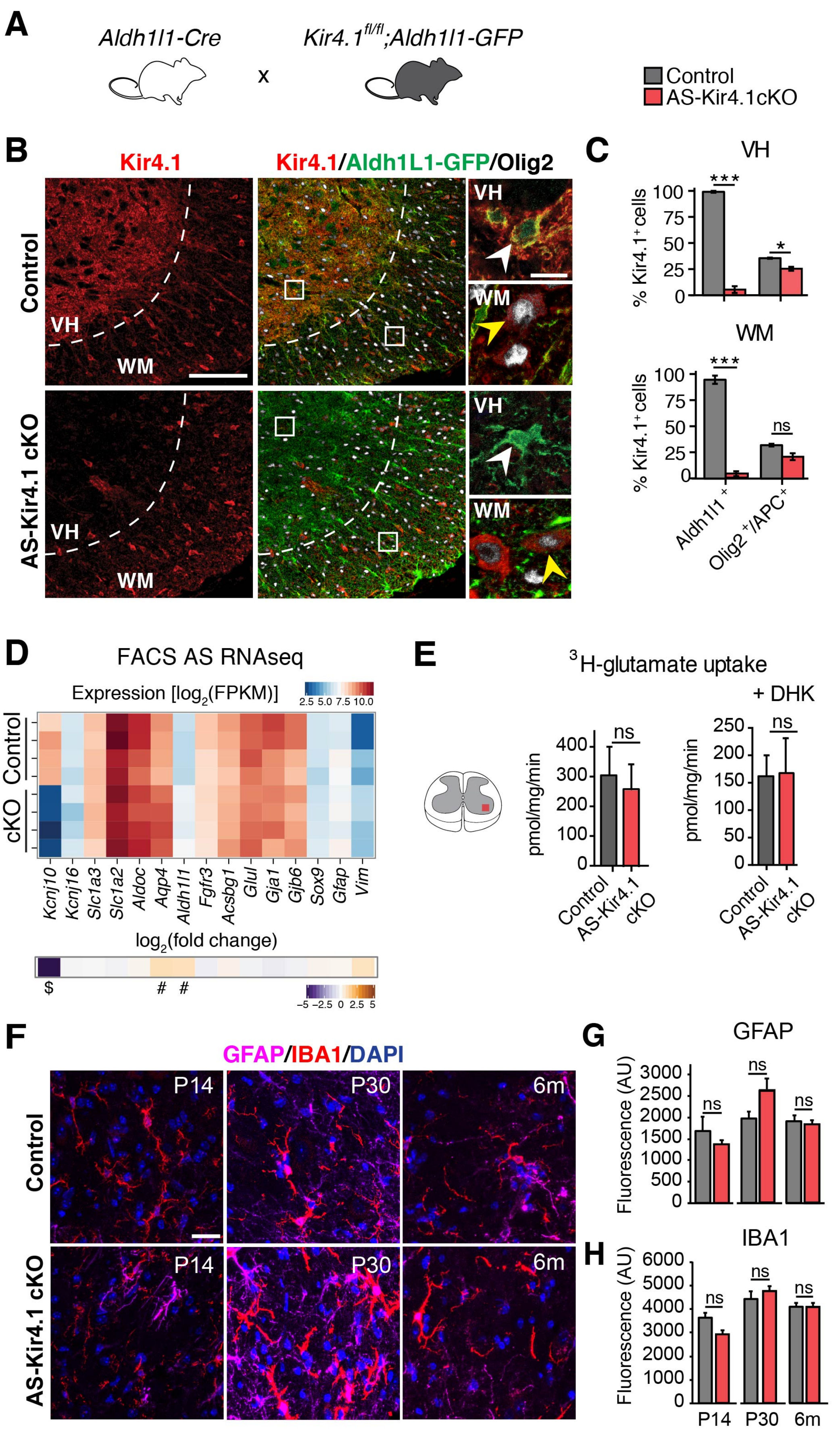
A
Aldh1l1-Cre
x
Kir4.1fl/fl;Aldh1l1-GFP
Control
AS-Kir4.1cKO
B
Kir4.1
Kir4.1/Aldh1L1-GFP/Olig2
Control
AS-Kir4.1 cKO
VH
WM
C
VH
WM
% Kir4.1+cells
Aldh1l1+
Olig2+/APC+
***
*
ns
D
FACS AS RNAseq
Expression [log2(FPKM)]
Control
cKO
Kcnj10 Kcnj16 Slc1a3 Slc1a2 Aldoc Aqp4 Aldh1l1 Fgfr3 Acsbg1 Glul Gja1 Gjb6 Sox9 Gfap Vim
log2(fold change)
$ # #
E
3H-glutamate uptake
+ DHK
pmol/mg/min
Control
AS-Kir4.1 cKO
F
GFAP/IBA1/DAPI
P14
P30
6m
G
GFAP
H
IBA1
Fluorescence (AU)
P14 P30 6m

Figure S4 | Characterization of *AS Kir4.1 cKO* mice. Related to Figure 2. A) Astrocyte-specific *Kir4.1* KO (*AS-Kir4.1cKO*) and cre-negative control animals were generated by crossing *Aldh1l-cre* and *Kir4.1$^{fl/fl}$-Aldh1l1-GFP* mice. **B**) Loss of Kir4.1 expression in *Aldh1l1-GFP*$^{+}$ AS mice but not Olig2^{+} oligodendrocytes in *AS-Kir4.1cKO* lumbar spinal cord at P30. Dotted line denotes gray/white matter boundary. VH: ventral horn. WM: white matter. Right: High-magnification AS and oligodendrocytes (scale bar: 100μm, insert: 10μm). **C)** Histological counts of Kir4.1 percent positive AS (Aldh1l1^{+}) and oligodendrocytes (Olig2^{+}/APC^{+}) in VH (top panel) and WM (bottom panel) lumbar spinal cord at P14 for AS (n = 4 mice) and P30 for oligodendrocytes (n =3 mice, mean ± sem, Welch's t-test). **D)** Top: Heat map showing mRNA expression levels of canonical AS markers in FACS-isolated AS from P14 *AS Kir4.1 cKO* and control mice. Bottom: Heat map of log2 fold changes of the indicated genes ($ and # denote significance). **p* < 0.05, *** *p* < 0.001, # $p < 10^{-6}$, $ $p < 10^{-100}$. **E)** Glutamate uptake is not altered in *AS Kir4.1 cKO* mice. Total (right) and GLT-1 mediated (left) uptake was measured using the ^{3}H-glutamate uptake assay on spinal cord samples from adult *AS-Kir4.1cKO* and cre-negative control animals (n=4-6 mice/group, mean ± sem, Mann-Whitney test). **F)** Absence of AS (GFAP) and microglia (IBA1) reactivity in the spinal cord of *AS Kir4.1 cKO* at all time points analyzed. **G, H)** Quantification of GFAP (**G**) and IBA1 (**H**) fluorescence levels in the ventral horn of *AS Kir4.1 cKO* and control animals at P14, P30 and 6m (n=3 mice/group, mean ± sem, Mann-Whitney test).

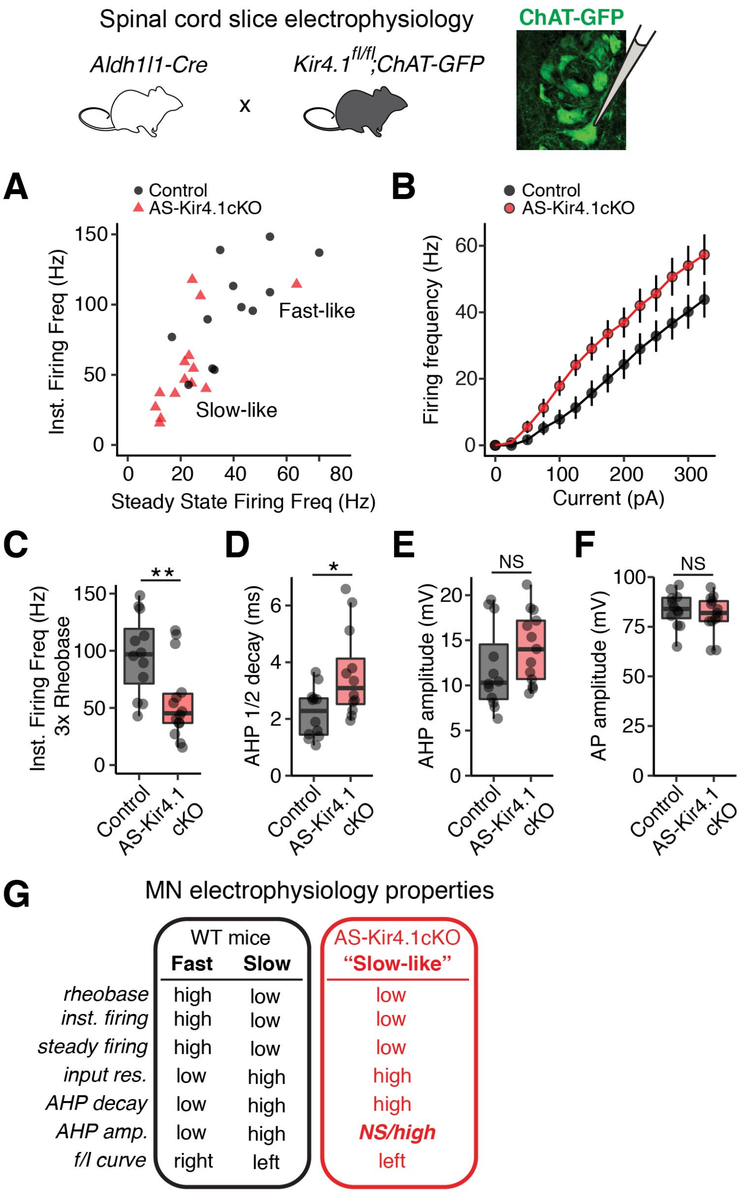

Figure S5 |AS *Kir4.1* is required for FαMN physiological properties. Related to Figure 3. (A) Scatter plot of MN instantaneous and steady state firing frequency calculated at three times rheobase. (**B)** MN firing frequency at 25 picoampere (pA) depolarizing current steps (n = 12 control MNs, n = 14 *AS-Kir4.1cKO* MNs from at least 3 animals per group, mean ± sem). (**C-F)** Instantaneous firing frequency calculated at three times rheobase (**C**), afterhyperpolarization (AHP) half decay time (**D**), AHP amplitude (**E**), and action potential (AP) amplitude (**F**) from *AS-Kir4.1cKO-ChAT-GFP* animals at P12-15 (n = 12 control MNs, n = 14 *AS-Kir4.1cKO* MNs from at least 3 animals per group, boxplot, Mann-Whitney test). Edges of boxplots denote interquartile range (25th – 75th percentile) with whiskers denoting 1.5 times the interquartile range and black line denoting the median value. * $p < 0.05$. (**G**) Table

highlighting differences in MN electrophysiological profile between fast and slow MNs identified in previous studies (left) (Hadzipasic et al., 2014; Muller et al., 2014) as compared to our findings from *AS-Kir4.1cKO* MNs (right).

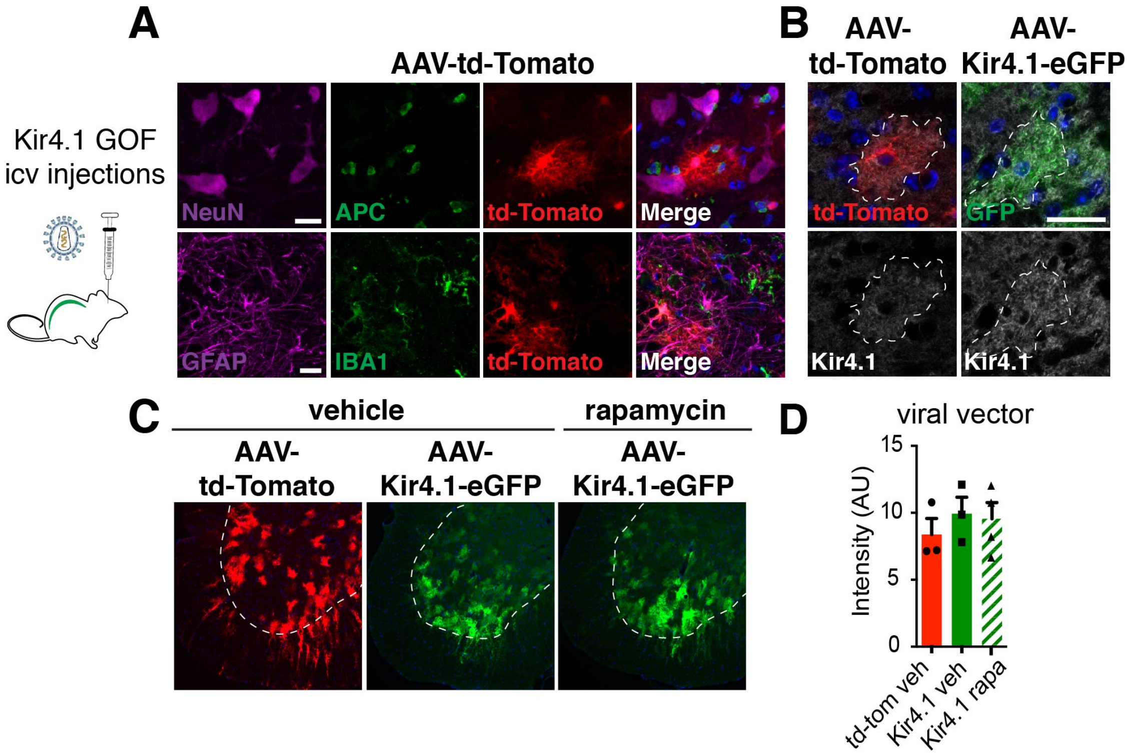

Figure S6 | Characterization of viral-mediated Kir4.1 overexpression in spinal cord AS. Related to Figure 5. A) Validation of AAV2/9-gfaABC$_1$D vector tropism in the mouse spinal cord. AAV-encoded td-Tomato is expressed in GFAP$^+$ AS but not in NeuN$^+$ neurons, IBA1$^+$ microglia or APC$^+$ oligodendrocytes in P30 mice (1 month post-injection). **B)** Increased Kir4.1 expression in AAV-Kir4.1-eGFP-injected mice as compared to AAV-td-Tomato controls. **C)** Representative images of viral transduction in the ventral horn of mice injected with AAV-td-tomato or AAV-Kir4.1-eGFP and treated with vehicle (left) or rapamycin (right). **D)** Rapamycin treatment does not alter viral transduction in the ventral horn (n=3 mice/group, mean ± sem, Mann-Whitney test).

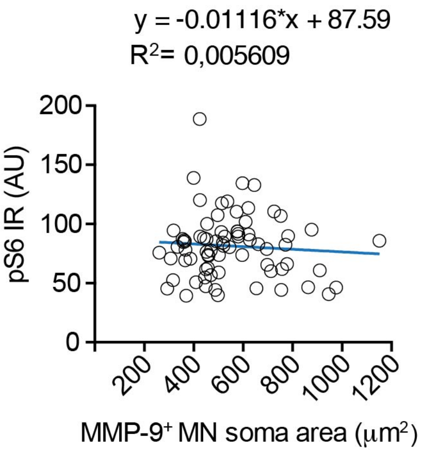

Figure S7 | Absence of correlation between pS6 fluorescence intensity and MN soma area. Related to Figure 6. pS6 IR value and corresponding soma area for individual MMP-9$^+$ MN in the spinal cord of control (cre negative) mice.

Patient #	Age (years)	Gender	Cause of death
1	86	female	Cerebrovascular ischemia
2	88	female	Bronchopneumonia
3	91	female	Cerebrovascular ischemia

Table S1. Clinical and pathological characteristics of spinal cord control patients. Related to Figure 1.

Patient #	Mutation	Age (years)	Gender	Source
Control No. 1	None	64	Male	Coriell ND41866*C
Control No. 2	None	78	Male	PMC4550814
Control No. 3 (isogenic)	None ($SOD1^{D90D}$)	50	Female	Su-Chun Zhang’s lab
SOD1 No. 1	$SOD1^{D90A}$	50	Female	Su-Chun Zhang’s lab
SOD1 No. 2	$SOD1^{D90A}$	70	Female	Coriell ND35664

Table S2. Human iPSC lines. Related to Figure 4.
