# Supplementary material for: Kir4.1-Dependent Astrocyte-Fast Motor Neuron Interactions Are Required for Peak Strength
Source: Neuron. 2018 Apr 18;98(2):306–319.e7. doi: 10.1016/j.neuron.2018.03.010 (PMC5919779; doi:10.1016/j.neuron.2018.03.010)
Supplement: Document S2. Article plus Supplemental Information [file mmc2.pdf]

Neuron

Article

Kir4.1-Dependent Astrocyte-Fast Motor Neuron Interactions Are Required for Peak Strength

Highlights

- Kir4.1 is upregulated in astrocytes around high-activity alpha motor neurons (MNs)
- Astrocyte *Kir4.1* KO caused decreased peak strength without alpha MN loss
- ALS patient-derived astrocytes show cell-autonomous Kir4.1 downregulation
- Astrocyte Kir4.1 regulates MN size through PI3K/mTOR/pS6 activation

Authors

Kevin W. Kelley, Lucile Ben Haim, Lucas Schirmer, ..., Andras Lakatos, Erik M. Ullian, David H. Rowitch

Correspondence

dhr25@medschl.cam.ac.uk

In Brief

Kelley et al. show that specialized astrocytes surrounding spinal cord fast α-motor neurons are critical to generate peak strength and that they are compromised by mutations in models of amyotrophic lateral sclerosis.

Kelley et al., 2018, Neuron *98*, 306–319
April 18, 2018 © 2018 The Authors. Published by Elsevier Inc.
https://doi.org/10.1016/j.neuron.2018.03.010

CellPress

Neuron
Article

Kir4.1-Dependent Astrocyte-Fast Motor Neuron Interactions Are Required for Peak Strength

Kevin W. Kelley,[1,2,10] Lucile Ben Haim,[1,2,10] Lucas Schirmer,[1,2] Giulia E. Tyzack,[3,4] Michaela Tolman,[5] John G. Miller,[6] Hui-Hsin Tsai,[1,2] Sandra M. Chang,[1,2] Anna V. Molofsky,[6] Yongjie Yang,[5] Rickie Patani,[3,4] Andras Lakatos,[7] Erik M. Ullian,[8] and David H. Rowitch[1,2,9,11,*]
[1]Eli and Edythe Broad Center of Regeneration Medicine and Stem Cell Research, University of California, San Francisco, San Francisco, CA 94143, USA
[2]Departments of Pediatrics and Neurosurgery, University of California, San Francisco, San Francisco, CA 94143, USA
[3]Department of Molecular Neuroscience, Institute of Neurology, University College London, London WC1N 3BG, UK
[4]The Francis Crick Institute, London NW1 1AT, UK
[5]Sackler School of Biomedical Sciences, Tufts University, Boston, MA 02111, USA
[6]Department of Psychiatry, University of California, San Francisco, San Francisco, CA 94143, USA
[7]John van Geest Centre for Brain Repair and Department of Clinical Neurosciences, University of Cambridge, Cambridge CB20QQ, UK
[8]Department of Ophthalmology, University of California, San Francisco, San Francisco, CA 94143, USA
[9]Department of Paediatrics and Wellcome Trust-MRC Cambridge Stem Cell Institute, University of Cambridge, Cambridge CB20QQ, UK
[10]These authors contributed equally
[11]Lead Contact
*Correspondence: dhr25@medschl.cam.ac.uk
https://doi.org/10.1016/j.neuron.2018.03.010

SUMMARY

Diversified neurons are essential for sensorimotor function, but whether astrocytes become specialized to optimize circuit performance remains unclear. Large fast α-motor neurons (FαMNs) of spinal cord innervate fast-twitch muscles that generate peak strength. We report that ventral horn astrocytes express the inward-rectifying K^+ channel Kir4.1 (a.k.a. *Kcnj10*) around MNs in a VGLUT1-dependent manner. Loss of astrocyte-encoded *Kir4.1* selectively altered FαMN size and function and led to reduced peak strength. Overexpression of Kir4.1 in astrocytes was sufficient to increase MN size through activation of the PI3K/mTOR/pS6 pathway. Kir4.1 was downregulated cell autonomously in astrocytes derived from amyotrophic lateral sclerosis (ALS) patients with SOD1 mutation. However, astrocyte Kir4.1 was dispensable for FαMN survival even in the mutant SOD1 background. These findings show that astrocyte Kir4.1 is essential for maintenance of peak strength and suggest that Kir4.1 downregulation might uncouple symptoms of muscle weakness from MN cell death in diseases like ALS.

INTRODUCTION

Astrocytes (AS) carry out general functions in the central nervous system (CNS), including blood-brain barrier formation, regulation of synaptogenesis, and the maintenance of metabolic, ionic, and neurotransmitter homeostasis (Allen, 2014; Allen and Barres, 2009; Matyash and Kettenmann, 2010). Because AS are pervasive throughout the CNS and their processes tile within domains, they are key environmental determinants for neural circuits. We have proposed that regionally diversified AS could become "optimized" to enhance local function (Freeman and Rowitch, 2013; Molofsky et al., 2012). Indeed, differences in AS morphology (Oberheim et al., 2012) and transcriptional profiles (Cahoy et al., 2008; Doyle et al., 2008; Zhang et al., 2014) suggest that AS comprise distinct classes with potentially varied regional activities (Ben Haim and Rowitch, 2017; Khakh and Sofroniew, 2015). Coordination of voluntary movement is complex and requires diversified motor neuron (MN) subtypes that form region- and muscle-specific interactions (Kanning et al., 2010). Pattern formation underlies generation of MN diversity (Stifani, 2014), and a similar region-restricted developmental mechanism is associated with specification and regional allocation of AS (Hochstim et al., 2008; Muroyama et al., 2005; Tsai et al., 2012). However, the relationship between local AS specialization, neuron subtype selective support, and neural circuit function remains poorly understood.

Spinal cord αMNs fall into two broad classes along the anterior posterior axis. Slow αMNs (SαMNs) innervate type I muscle fibers and generate fatigue-resistant, low-force contractions while fast αMNs (FαMNs) project to type II fast-fatigable muscle fibers and produce brief, but high force outputs (Burke et al., 1971; Eccles et al., 1957). Gamma MNs (γMNs) innervate muscle spindles to ensure proper muscle fiber tension. The morphological, transcriptional, and biophysical properties of these MN subtypes match their corresponding muscle contractile properties (Henneman, 1957; Kernell, 1966; Müller et al., 2014). FαMNs are larger in size, are activated at higher thresholds, and fire action potentials in high-frequency

This is an open access article under the CC BY license (http://creativecommons.org/licenses/by/4.0/).

bursts that are readily distinguishable from SαMNs (Hadzipasic et al., 2014).

Amyotrophic lateral sclerosis (ALS) is a neurodegenerative disease characterized by progressive muscle weakness and paralysis resulting from MN death (Cleveland and Rothstein, 2001). Although the majority of cases are sporadic, familial forms of ALS have provided insight into genetic causes of the disease (Robberecht and Philips, 2013). Of these, constitutive mutations of superoxide dismutase 1 (SOD1) (Rosen, 1993) have been the most extensively studied and are sufficient to cause clinical symptoms and MN death in rodent models (Philips and Rothstein, 2015). FαMNs innervating fast-fatigable muscle fibers are selectively vulnerable in ALS (Saxena and Caroni, 2011). In SOD1G93A transgenic ALS mice, these MNs display early transcriptional alterations and endoplasmic reticulum (ER) stress (Saxena et al., 2009) and selectively express matrix metalloproteinase-9 (MMP-9), which regulates FαMN survival and disease progression (Kaplan et al., 2014). Indeed, FαMN cell death in ALS is generally thought to cause loss of peak strength and clinical decline in both rodent models and human patients (Kanning et al., 2010; Kaplan et al., 2014; Pun et al., 2006). MN death in ALS also involves non-cell-autonomous mechanisms due to glial cell dysfunction (Lobsiger and Cleveland, 2007). For instance, selective removal of SOD1G37R in microglia (Boillée et al., 2006), oligodendrocyte precursor cells (Kang et al., 2013), and AS (Yamanaka et al., 2008) slowed disease progression, suggesting that mutant SOD1 is detrimental to glial cell functions.

Is there a role for local ventral horn AS to selectively maintain the physiological properties and function of MN subtypes, and is this role disrupted in ALS? We have previously shown that ventral horn AS-encoded function of *Sema3a* is essential for the survival of αMNs (Molofsky et al., 2014). Here, we focused on the astrocytic inward-rectifying K^+ channel Kir4.1 (a.k.a. *Kcnj10*), which is selectively enriched in ventral compared to dorsal spinal cord AS (Olsen et al., 2007). Kir4.1 and Kir5.1 are the major inward-rectifying K^+ channels expressed in AS and are important for K^+ homeostasis, establishing the characteristic high resting K^+ current and setting AS resting membrane potential (Djukic et al., 2007; Olsen et al., 2006; Seifert et al., 2009; Sibille et al., 2015). Downregulation of AS Kir4.1 has been observed in several CNS diseases such as acute and chronic neuroinflammation (Schirmer et al., 2014; Zurolo et al., 2012), spinal cord injury (Olsen et al., 2010), and transgenic mouse models of ALS (Bataveljić et al., 2012; Kaiser et al., 2006), raising the question of whether such dysregulation is pathological or maladaptive. Here, we report that AS Kir4.1 is specifically required to maintain functional properties—but not survival—of FαMN populations in the mouse spinal cord. AS Kir4.1 expression surrounding FαMNs was developmentally upregulated in a VGLUT1-dependent manner. Conditional knockout (cKO) of AS-encoded *Kir4.1* (*AS-Kir4.1cKO*) led to a highly selective and pervasive reduction in FαMN size and characteristic electrophysiological properties. *AS-Kir4.1cKO* animals showed decreased fast-twitch muscle fiber size and peak strength. In contrast, SαMNs and γMNs showed no detectable abnormalities. Viral overexpression of Kir4.1 in AS was sufficient to increase both FαMN and SαMN soma size through activation of the PI3K/mTOR/pS6 pathway. In cultured AS derived from ALS patient-induced pluripotent stem cells (iPSCs), we observed significant reduction of KIR4.1 (*KCNJ10*) expression, suggesting that ALS-associated SOD1 mutation causes cell-autonomous downregulation of KIR4.1. Because AS Kir4.1 was dispensable for FαMN survival even in the mutant SOD1 ALS mice, we conclude that its function is required to maintain peak strength, FαMN cellular, and biophysical properties, but not MN survival. Collectively, these results suggest that clinical loss of peak strength in ALS could signify downregulation of AS Kir4.1 expression and therefore be uncoupled from FαMN cell death.

RESULTS

Kir4.1 Upregulation in Ventral Horn AS Surrounding FαMNs

To identify regions with the highest *Kir4.1* expression levels, we analyzed mRNA expression profiling data from human (GTEx-Consortium, 2015) and mouse (Kasukawa et al., 2011) CNS. *Kir4.1* transcript levels varied by more than 5-fold across CNS regions, with the highest expression in the spinal cord of both humans and mice (Figures 1A and 1B), consistent with previous studies (Nwaobi et al., 2016; Olsen et al., 2007). The spinal cord has well-defined organization in the dorsoventral (DV) axis with MNs located in the ventral horn (Figures 1C and 1D). Kir4.1 showed marked expression in ventral gray matter as compared to dorsal horn in human (Figure 1C; Table S1) and mouse (Figures 1D and 1E) spinal cord. In mice, *Kir4.1* was enriched in ventral versus dorsal AS in both cultured and fluorescence-activated cell sorting (FACS)-purified AS from *Aldh1l1-GFP* mice (Cahoy et al., 2008; Tien et al., 2012) (Figure 1F).

We determined the cell-type contributions to Kir4.1 expression in the mouse spinal cord using *Aldh1l1-GFP* mice to label AS along with markers of oligodendrocytes and neurons. We found an 8-fold enrichment of *Kir4.1* mRNA in FACS-isolated AS compared to non-AS (Figure 1G), consistent with mRNA expression profiling data from human and mouse cortex (Figures S1A and S1B). Kir4.1 was also expressed in approximately 30% of gray matter oligodendrocytes in the ventral horn, but not in $NeuN^+$ or choline acetyltransferase $(ChAT)^+$ neurons (Figure 1D; Figures S1C and S1D). Using MMP-9 as a marker of FαMNs (Kaplan et al., 2014), we observed a graded expression pattern of Kir4.1 with highest levels surrounding MMP-9^+ FαMNs as compared to MMP-9^- SαMNs (Figures 1H and 1I).

As Kir4.1 function is related to synaptic activity (Cheung et al., 2015) and neuronal signaling regulates the expression levels of other AS transporters (Muthukumar et al., 2014; Yang et al., 2009), we investigated Kir4.1 expression in relation to the excitatory presynaptic terminal markers VGLUT1 (Figures S2A–S2D) and VGLUT2 (Figure S2E) in the developing mouse spinal cord. We observed correlated spatiotemporal expression of Kir4.1 with VGLUT1, but not VGLUT2, expression during development in the mouse spinal cord (Figure S2). Interestingly, FαMNs have a larger VGLUT1 synaptic density than SαMNs (Basaldella et al., 2015), consistent with the observed higher Kir4.1 expression levels around FαMNs (Figures 1H and 1I). To determine whether VGLUT1 activity is required for AS Kir4.1 expression, we analyzed *VGLUT1*$^{-/-}$ conventional KO animals (Morel et al., 2014). We found that *VGLUT1* loss of function led to dramatic

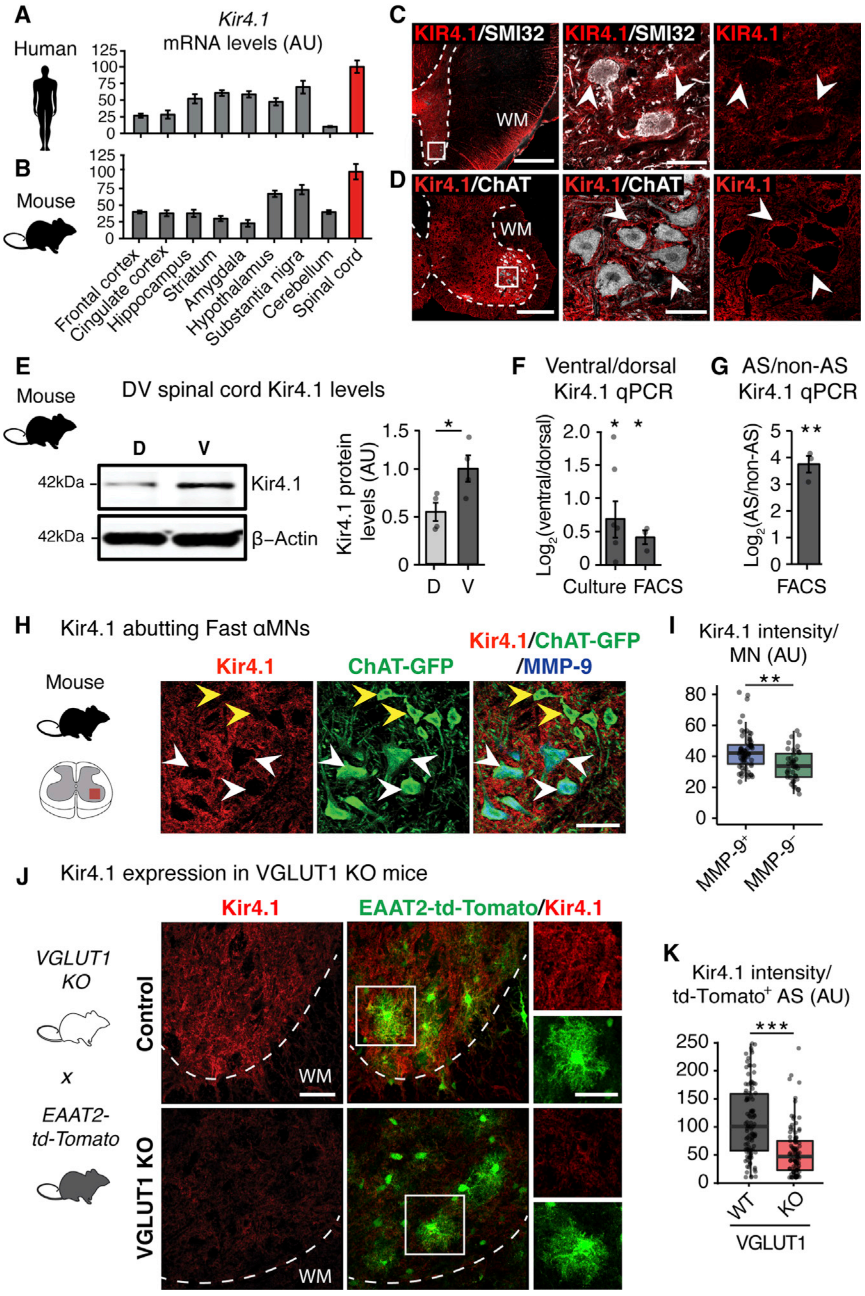

Figure 1. Kir4.1 Upregulation in Ventral Horn AS around Large FαMN MNs

(A and B) *Kir4.1* mRNA is enriched in spinal cord compared to other CNS regions in humans (GTEx-Consortium, 2015) (A) and mice (Kasukawa et al., 2011) (B). Data are represented as mean ± SEM.

(C and D) Perineuronal enrichment of Kir4.1 expression in human and mouse ventral spinal cord.

(C) KIR4.1 is expressed around SMI32^{+} neurons in adult human ventral spinal cord (scale bars, 1 mm, left; 50 μm, right). Representative image of n = 3 control human spinal cord. Patient data are provided in Table S1.

(D) Kir4.1 is expressed around ChAT^{+} MNs in mouse ventral lumbar spinal cord at P16 (scale bars, 200 μm, left; 50 μm, right). Dotted line denotes gray/white matter boundary. WM, white matter. Arrowheads denote Kir4.1 enrichment around ventral horn neurons. High-resolution images are single-plane confocal images.

(E) Increased ventral (V) compared to dorsal (D) Kir4.1 protein by western blot from mouse lumbar spinal cord at P30 (n = 4 mice, mean ± SEM, Welch's t test).

(F) Left: fold change of *Kir4.1* mRNA levels between ventral and dorsal samples from cultured (n = 6 mice, mean ± SEM, one-sample t test) neonatal mouse spinal cord AS. Right: FACS-purified AS from P5 *Aldh1l1-GFP*$^{+}$ (n = 3 mice, mean ± SEM, one-sample t test) mouse spinal cord.

(G) *Kir4.1* mRNA levels in P5 *Aldh1l1-GFP*$^{+}$ FACS-purified AS compared to *Aldh1l1-GFP*$^{-}$ non-AS cells from mouse ventral spinal cord (n = 3 mice, mean ± SEM, one-sample t test).

(H) Kir4.1 protein is preferentially found around larger MMP-9^{+} FαMNs (white arrowheads) compared to smaller MMP-9^{-} SαMNs (yellow arrowheads) at P30 (scale bar, 50 μm).

(I) Quantification of Kir4.1 signal intensity around individual MMP-9^{+} or MMP-9^{-} MN (n = 4 mice, >100 MN counts/animal, boxplot, Mann-Whitney test).

(J) Kir4.1 loss in AS from *VGLUT1 KO* animals at P26. *VGLUT1 KO* mice were crossed with *EAAT2-td-Tomato* reporter for AS visualization.

(K) Quantification of Kir4.1 immunofluorescence intensity per AS (EAAT2-td-tomato^{+}) (n = 2 mice, >50 AS counts/animal, boxplot, Mann-Whitney test; scale bar, 40 μm, insert: 20 μm). $^{*}p < 0.05$, $^{**}p < 0.01$, $^{***}p < 0.001$. Edges of boxplots denote interquartile range (25th–75th percentile) with whiskers denoting 1.5 times the interquartile range and the black line denoting the median value.

reduction of AS Kir4.1 expression in ventral horn (Figures 1J and 1K). Consistent with Kir4.1 expression dictated in part by neuron-derived factors, we found that spinal cord *Kir4.1* (*Kcnj10*) levels were downregulated in a genetic MN ablation model at embryonic day 18.5 (E18.5) (Figure S3). Together, these findings suggest that AS Kir4.1 expression levels show both regional (i.e., ventral versus dorsal horn) and subregional (i.e., FαMNs versus SαMNs) differences and are regulated in a VGLUT1-dependent manner *in vivo*.

AS-Encoded *Kir4.1* Function Is Dispensable for MN Survival but Required for Characteristic FαMN Morphology

The findings above suggested that AS Kir4.1 developmental upregulation requires VGLUT1 synaptic signaling on MNs. To investigate a requirement for AS-encoded *Kir4.1* for ventral horn MNs, we intercrossed *Aldh1l1-cre*, which targets most AS in the ventral spinal cord (but not MNs, and only 30% of oligodendrocytes; Molofsky et al., 2014; Tien et al.,

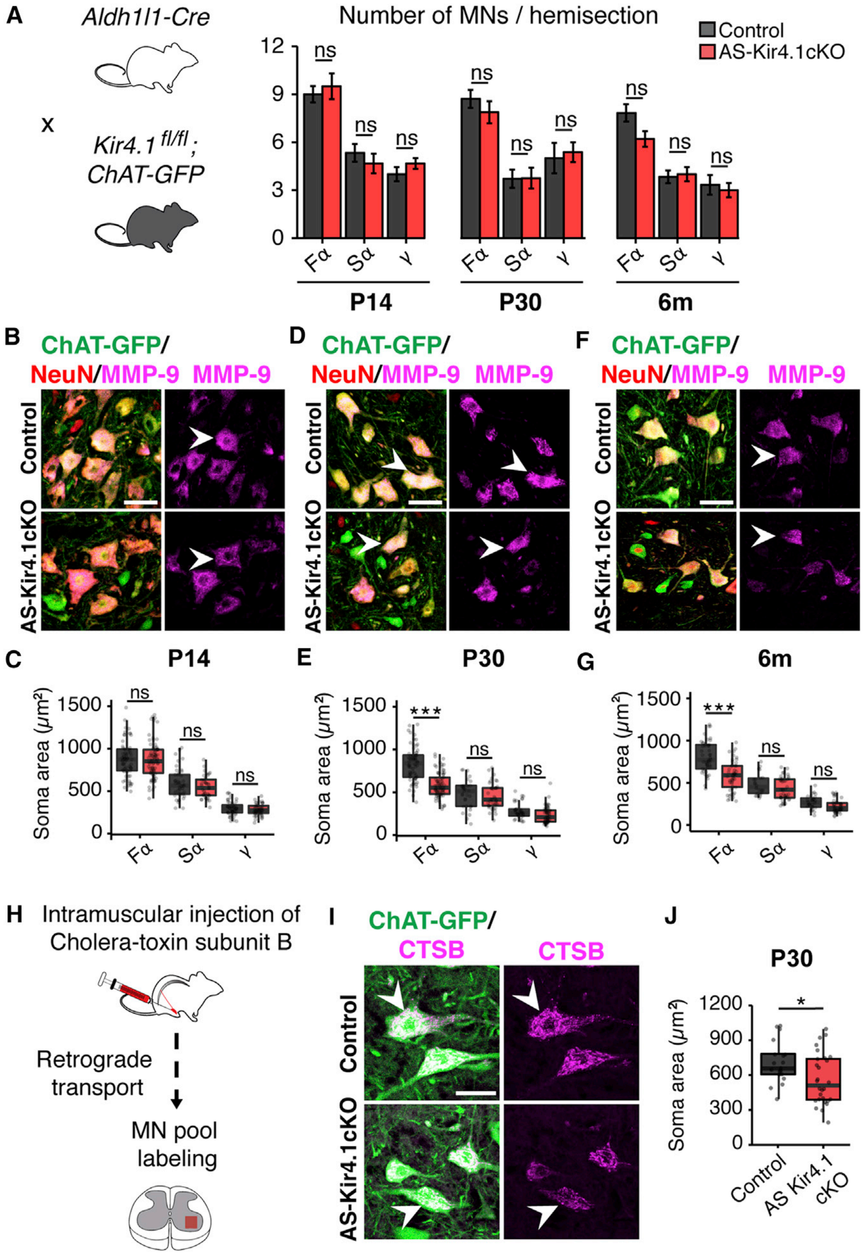

Figure 2. AS *Kir4.1* Is Required for Maintenance of FαMN Size

(A) Left: breeding scheme. *AS-Kir4.1cKO* and cre-negative control animals were bred with *ChAT-GFP* mice for MN visualization. Right: total FαMN ($ChAT^+$/$MMP\text{-}9^+$/$NeuN^+$), SαMN ($ChAT^+$/$MMP\text{-}9^-$/$NeuN^+$), and γMN ($ChAT^+$/$MMP\text{-}9^-$/$NeuN^-$) numbers are equivalent in *AS-Kir4.1cKO* and cre-negative control mice at the indicated ages (n = 3 mice/group, mean ± SEM, lumbar spinal cord, Welch's t test).

(B, D, and F) Representative images of lumbar MNs at P14 (B), P30 (D), and 6 months (F). Arrowheads denote example FαMNs (scale bar, 50 μm).

(C, E, and G) Quantification of MN size at P14 (C), P30 (E), and 6 months (G) (n = 3 mice/group, >100 MN counts/animal, boxplot, Mann-Whitney test).

(H) Schematic of retrograde labeling of MN pools using intramuscular injection of fluorescent cholera toxin subunit B (CTSB) in the tibialis anterior (TA) muscle.

(I) Representative images of retrograde-labeled ventral horn MNs in *AS-Kir4.1cKO* and cre-negative control mice. Arrowheads denote example putative FαMNs (scale bar, 50 μm).

(J) Quantification of $ChAT\text{-}GFP^+CTSB^+$ MN soma area from (G) (n = 3 mice/group, >50 MN counts/animal, boxplot, Mann-Whitney test). *$p < 0.05$, ***$p < 0.001$. Edges of boxplots denote interquartile range (25th–75th percentile) with whiskers denoting 1.5 times the interquartile range and black line denoting the median value.

2012), with a conditional floxed allele of *Kir4.1* (Djukic et al., 2007) (Figures S4A–S4C). *Aldh1l1-cre:Kir4.1^fl/fl* animals, heretofore called *AS-Kir4.1cKO*, survived in normal numbers up to at least 1 year of age (data not shown). Mice were crossed with *Aldh1l1-GFP* reporter animals for AS visualization. AS Kir4.1 protein expression was undetectable in ventral horn gray matter AS of *AS-Kir4.1cKO* mice compared to cre-negative controls (Figures S4A–S4C); in contrast, Kir4.1 expression was preserved in 70% of oligodendrocytes in *AS-Kir4.1cKO* animals (Figures S4A–S4C). The number of *Aldh1l1-GFP*$^+$ AS was identical in *AS-Kir4.1cKO* and cre-negative control mice (data not shown). RNA sequencing (RNA-seq) of FACS-purified spinal cord *Aldh1l1-GFP*$^+$ AS from *AS-Kir4.1cKO* and cre-negative control mice showed that: (1) *Kir4.1* (*Kcnj10*) was the most significantly downregulated transcript, whereas in contrast, (2) other AS differentiation genes, including glutamate transporters, showed non-significant changes (Figure S4D). Although previous work showed that Kir4.1-dependent maintenance of AS membrane potential was required for glutamate transporter-1 (GLT-1) function (Djukic et al., 2007), we did not find any difference in total and GLT-1-mediated (+DHK) glutamate uptake between *AS-Kir4.1cKO* and cre-negative control spinal cords (Figure S4E). These findings indicate that *Aldh1l1-cre* drives AS loss of *Kir4.1* function but does not change glutamate uptake/transporter expression. Moreover, we did not observe gliosis or microglial activation at any time points analyzed in *AS-Kir4.1cKO* mice (Figures S4F–S4H).

Having confirmed AS *Kir4.1* loss of function, we next focused on consequences for MN survival. *AS-Kir4.1cKO* and cre-negative controls were intercrossed with *ChAT-GFP* mice for MN visualization (Figure 2A). We did not detect losses in FαMN, SαMN, or γMN populations in the lumbar spinal cord of *AS-Kir4.1cKO* mice compared to cre-negative

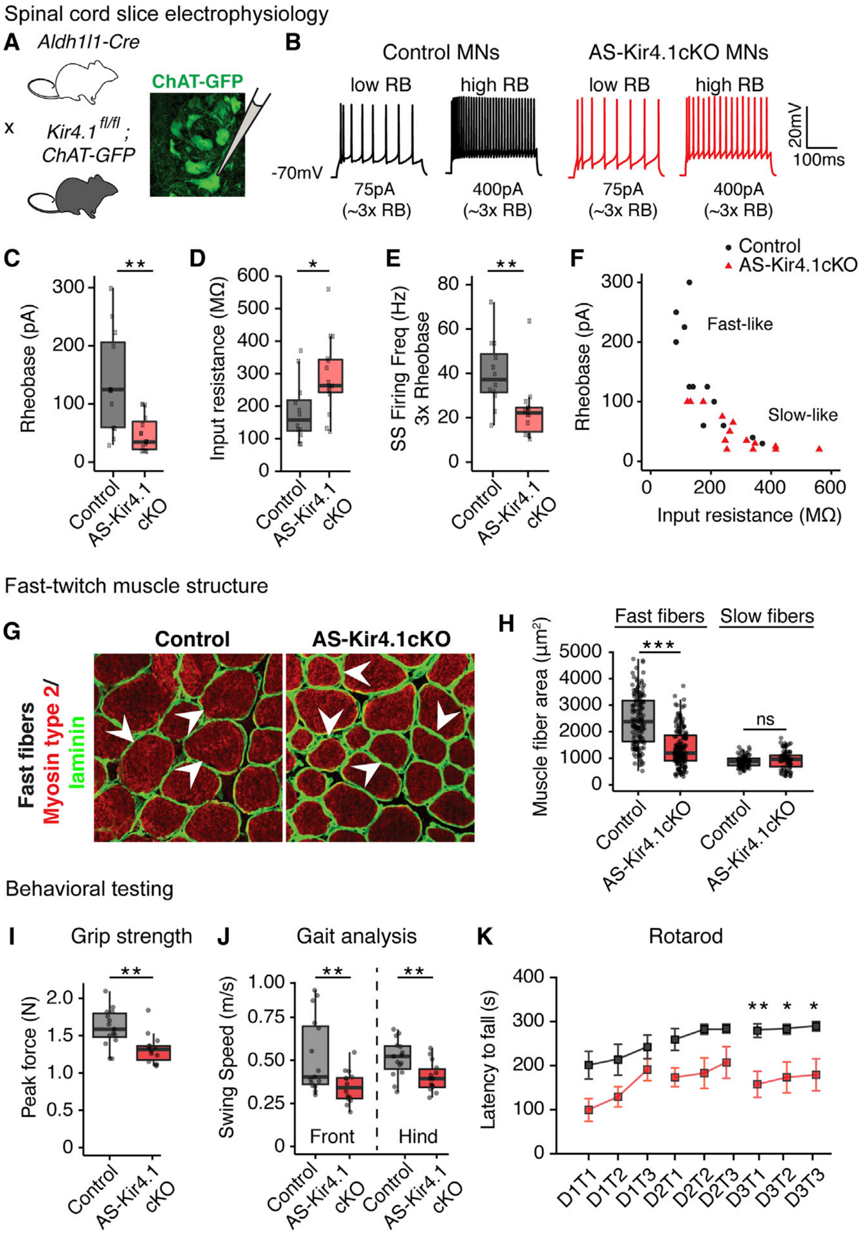

Figure 3. AS *Kir4.1* Is Required for FαMN Function, Fast-Twitch Muscle Fiber Size, and Peak Force

(A) Breeding scheme and electrophysiology recording schematic.

(B) Two representative current steps at 3× rheobase (RB).

(C–E) Rheobase (C), input resistance (D), and steady-state (SS) firing frequency (at 3× rheobase) (E) demonstrate altered intrinsic electrophysiological deficits in MNs from *AS-Kir4.1cKO-ChAT-GFP* animals at P12–P15 (n = 12 control MNs, n = 14 *AS-Kir4.1cKO* MNs from at least 3 animals per group, boxplot, Mann-Whitney test).

(F) Input resistance versus rheobase scatterplot shows shift in electrophysiological properties from fast-like to slow-like in *AS-Kir4.1cKO* compared to control MNs.

(G) Cross sections of TA muscle fibers immunolabeled for marker of fast-twitch muscle (myosin type 2) and laminin in P30 *AS-Kir4.1cKO* and cre-negative control animals (scale bar, 50 μm).

(H) Quantification of TA muscle fiber cross-sectional area (n = 3 mice/group, >100 muscle fibers/animal, boxplot, Mann-Whitney test).

(I–K) Abnormal muscle strength behavior in *AS-Kir4.1cKO* mice. Adult *AS-Kir4.1cKO* animals generate less peak force (>P50, n = 14–15 mice/group, boxplot, Welch's t test) (I). *AS-Kir4.1cKO* animals have slower front and hindlimb movements as assessed by gait analysis with the catwalk behavioral test. Swing speed corresponds to the limb speed while in the air (same animals as in I, boxplot, Welch's t test) (J). *AS-Kir4.1cKO* animals display a shorter latency to fall on the accelerating rotarod at P30–P35 (n ≥ 7 mice/group, mean ± SEM, two-way ANOVA, Bonferroni post hoc test) (K). Mice performed three trials (T, T2, and T3) per day on 3 consecutive days (D1, D2, and D3). *$p < 0.05$, **$p < 0.01$. Edges of boxplots denote interquartile range (25th–75th percentile) with whiskers denoting 1.5 times the interquartile range and black line denoting the median value.

controls at postnatal day 14 (P14), P30, and 6 months of age, as shown in Figure 2A. Thus, AS Kir4.1 is dispensable for the specification and survival of MNs. We next analyzed the morphological properties of MN subpopulations (Kanning et al., 2010). While we observed normal mature soma sizes for all MN subtypes at P14 (Figures 2B and 2C), by P30 the largest $ChAT^+$ MN populations were no longer detectable in the lumbar (L3–L6) ventral spinal cord of *AS-Kir4.1cKO* mice. Indeed, we found a selective decrease in size of FαMNs at P30 and 6 months of age, whereas the size of SαMNs and γMNs remained unaffected (Figures 2B–2G). Retrograde labeling with cholera toxin subunit B (CTSB) of αMNs that innervate the tibialis anterior (TA) muscle, which contains primarily fast-twitch muscle fibers (Kaplan et al., 2014), confirmed reduced FαMN size in *AS-Kir4.1cKO* animals compared to cre-negative controls (Figures 2H–2J). Together, these findings indicate that the maintenance of large FαMN size has a selective dependence on AS Kir4.1.

Loss of Fast Electrophysiological Signature, Fast-Twitch Muscle Fiber Size, and Peak Strength in *AS-Kir4.1cKO* Mice

The pervasive expression of AS Kir4.1 in ventral horn predicted that loss of *Kir4.1* function would affect K^+ homeostasis in the region and alter physiologic function of all MN subtypes. To test this, we performed whole-cell patch-clamp recordings on MNs in acute lumbar spinal cord preparations from P12–P15 *AS-Kir4.1cKO* and cre-negative control animals intercrossed with *ChAT-GFP* mice for MN visualization (Figures 3A and 3B). FαMN electrophysiological properties can be

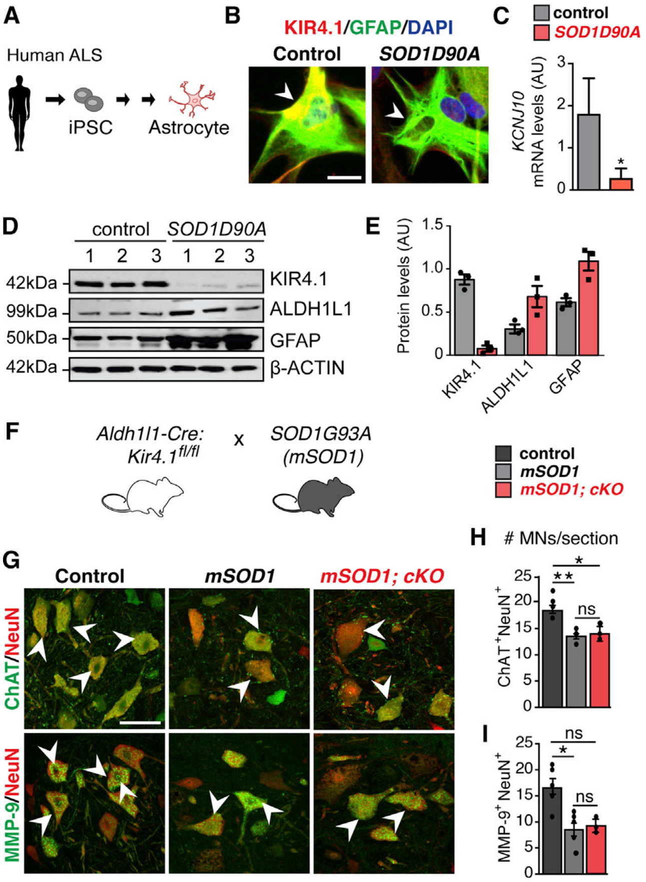

Figure 4. Cell-Autonomous AS *Kir4.1* Loss Does Not Alter MN Survival in ALS

(A) Schematic of iPSC-derived AS from human SOD1D90A ALS patients and non-ALS controls (patient data are provided in Table S2).

(B) Representative images of iPSC-derived AS from human SOD1D90A ALS patients and non-ALS controls labeled with KIR4.1 and GFAP (scale bar, 40 μm).

(C) *KCNJ10* mRNA levels are downregulated in SOD1G90A iPSC-derived AS as compared to controls (n = 2–3 independent cultures, mean ± SEM, Mann-Whitney test).

(D) Western blot of KIR4.1, ALDH1L1, and GFAP on iPSC-derived AS from human SOD1D90A ALS patients and non-ALS controls.

(E) Quantification of KIR4.1, ALDH1L1, and GFAP protein levels from western blot in (D) (n = 3/group, mean ± SEM, Mann-Whitney test).

(F) Breeding scheme used for the loss of function of AS *Kir4.1* in SOD1G93A (*mSOD1*) mutant background.

(G) Representative images of ventral horn lumbar spinal cord at P80 from cre-negative control, *mSOD1*, and *mSOD1*; *AS-Kir4.1cKO* (*mSOD1; cKO*) animals labeled with MN markers (scale bar, 50 μm).

(H and I) Quantification of ChAT$^+$NeuN$^+$ (H) and MMP-9$^+$NeuN$^+$ (I) MN numbers in cre-negative control, *mSOD1*, and *mSOD1; cKO* animals (n = 4–7 mice/group, >100 MN counts/animal, mean ± SEM, Kruskal-Wallis test). *$p < 0.05$, **$p < 0.01$.

classified with respect to SαMNs by their smaller input resistance, larger activation threshold (rheobase), shorter afterhyperpolarization (AHP) half-decay time, shorter AHP amplitude, and faster instantaneous and steady-state firing frequency (Hadzipasic et al., 2014; Müller et al., 2014). MNs from *AS-Kir4.1cKO* animals had a significantly lower activation threshold (rheobase), larger input resistance, decreased instantaneous and steady-state firing frequency, and increased AHP half-decay time (Figures 3C–3E; Figures S5C and S5D). Interestingly, these findings are consistent with FαMN physiological dysfunction and a shift toward "slow-like" properties (Figure 3F; Figures S5A and S5G). Similarly, analysis of instantaneous firing frequency and steady-state firing frequency (at 3× rheobase), which distinguishes slow and fast MN properties (Hadzipasic et al., 2014), showed that *AS-Kir4.1cKO* MNs are significantly more "slow-like" compared to control MNs (Figures S5A and S5C). *AS-Kir4.1cKO* MNs displayed a left shift of the current versus firing frequency curve in response to progressively increasing depolarizing currents (Figure S5B), which is also consistent with previously described "slow-like" MN properties (Müller et al., 2014). We did not observe significant differences in the AHP amplitude (Figure S5E) or in the action potential height (Figure S5F) or half-width (data not shown). These findings indicate that maintenance of many FαMN biophysical properties depends on intact AS Kir4.1 expression/function (Figure S5G).

As previous studies have suggested a relationship between MN area and the size of the corresponding muscle fiber subset (Kanning et al., 2010), we analyzed fast- and slow-twitch muscle fibers by histology. Indeed, we observed reduced fiber areas of the TA—a predominantly fast-twitch muscle—from *AS-Kir4.1cKO* mutants versus controls at P30 (Figures 3G and 3H). In contrast, slow-twitch muscle fiber size was unchanged in these animals (Figure 3H).

We next studied the behavioral consequences of AS *Kir4.1* deletion. Consistent with our observation that AS *Kir4.1* was dispensable for MN survival, *AS-Kir4.1cKO* adult animals did not develop paralysis even at later time points (>1 year of age). Because FαMNs are necessary for the generation of maximal force output (Müller et al., 2014), we used the grip strength test to measure peak force. We found that *AS-Kir4.1cKO* animals have decreased maximal peak force as compared with cre-negative controls (Figure 3I). Consistent with this, *AS-Kir4.1cKO* mice showed slower front and hindlimb movements (Figure 3J) and were unable to run the fast speeds necessary to complete the accelerating rotarod task (Figure 3K). However, *AS-Kir4.1cKO* mice displayed normal spontaneous locomotor activity and basic movements in the open field test (data not shown). Together, these results suggest that AS Kir4.1 is selectively required for behavioral tasks involving strength or fast movements.

Kir4.1 Expression Is Reduced in Human ALS AS and Dispensable for MN Survival in SOD1G93A ALS Mice

Previous work reported a progressive decrease of Kir4.1 expression in the spinal cord of SOD1G93A ALS mice (Kaiser et al., 2006), which exhibit a selective loss of FαMN populations (Kaplan et al., 2014; Pun et al., 2006). To ascertain whether SOD1 mutation is sufficient to decrease KIR4.1 expression in human AS, we investigated iPSC-derived AS from ALS patients and controls (Figure 4A; Table S2). As shown in Figures 4B–4E, we observed significantly decreased KIR4.1

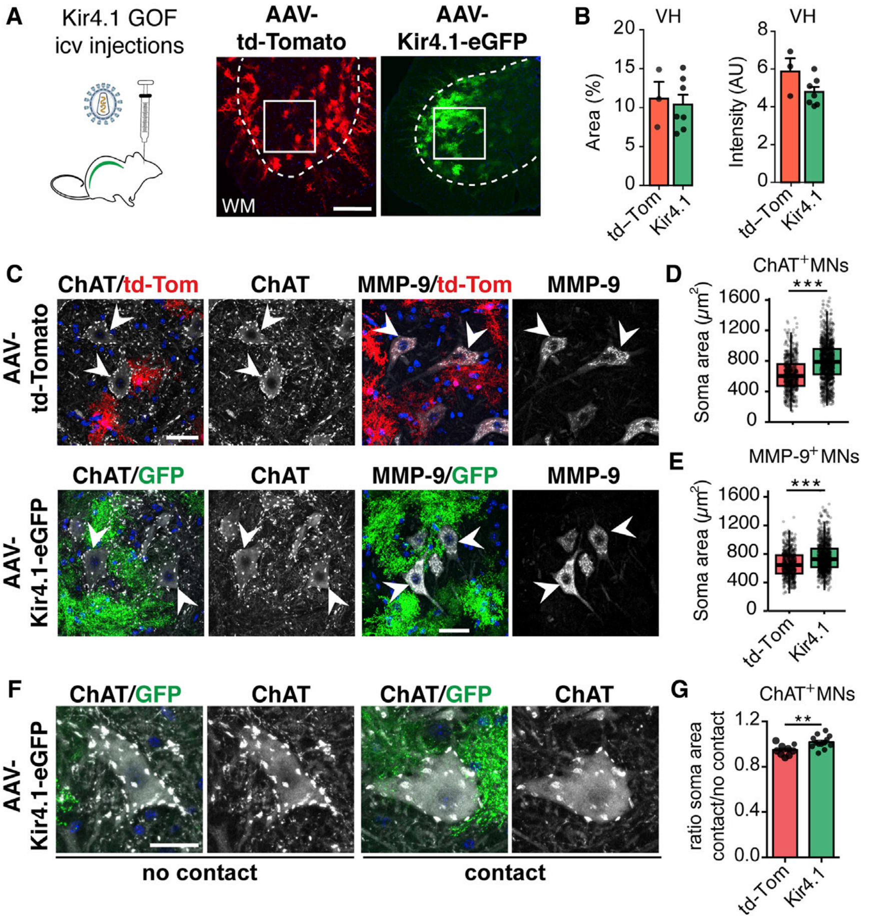

Figure 5. Kir4.1 Viral-Mediated Overexpression Is Sufficient to Increase MN Size

(A and B) Left: schematic of intracerebroventricular injections in neonatal mice (P2–P3) of AAV-encoding Kir4.1-eGFP or control td-Tomato vectors for gain-of-function (GOF) experiments (A). Right: viral transduction of ventral spinal cord with AAV-td-Tomato or AAV-Kir4.1-eGFP quantified in (B) (n = 3–7 mice/group, mean ± SEM, Mann-Whitney test).

(C) ChAT (right) and MMP-9 (left) immunofluorescent staining in the ventral spinal cord of AAV-td-Tomato and AAV-Kir4.1-eGFP-injected mice.

(D and E) Quantifications of ChAT^{+} (D) and MMP-9^{+} (E) MN soma area in P60 mice (2 months post injection) (n = 9–13 mice/group, 70–100 MNs counts/animal, boxplot, Mann-Whitney test, scale bar, 40 μm).

(F) Example of MNs in contact (right) or not (left) with Kir4.1-overexpressing AS.

(G) Ratio of soma area of MN contacting/non-contacting transduced AS (n = 9–13 mice/group, mean ± SEM, Mann-Whitney test). *p < 0.05, **p < 0.01, ***p < 0.001. Edges of boxplots denote interquartile range (25th –75th percentile) with whiskers denoting 1.5 times the interquartile range and black line denoting the median value.

expression in cultured human AS carrying the SOD1D90A mutation. KIR4.1 levels were downregulated both at the mRNA level as shown by qPCR (Figure 4C) and at the protein level showed by western blot (Figures 4D and 4E). Loss of KIR4.1 expression in human SOD1D90A mutant iPSC-AS appears selective, as ALDH1L1 and GFAP expression tended toward upregulation (Figures 4D and 4E). These findings in human patient-derived AS (in the absence of neurons) indicate that mutant SOD1 downregulates KIR4.1 in a cell-autonomous manner.

While *AS-Kir4.1cKO* did not cause MN cell death in wild-type (WT) background mice (see above), it was possible that AS Kir4.1 could influence cell loss in the more stressful setting of ALS. To rule out a requirement for AS Kir4.1 to maintain MN survival in an animal model of ALS, we assessed MN populations in WT, SOD1G93A (*mSOD1*), and *AS-Kir4.1cKO*, SOD1G93A (*mSOD1*, *cKO*) compound transgenic animals (Figure 4F). In keeping with prior reports (Vinsant et al., 2013), we observed MN loss in *mSOD1* mice at P80; however, we found no evidence for exaggerated losses in total MN or FαMN numbers in compound transgenic versus mice with SOD1G93A mutation alone (Figures 4G–4I). These findings indicate that AS Kir4.1 is dispensable for MN survival at P80 even in the setting of mutant SOD1G93A mutation.

Kir4.1 Overexpression in Spinal Cord AS Promotes Non-selective Increases in αMN Size

We next used an AS-selective gain-of-function approach in spinal cord to evaluate the effect of Kir4.1 on MN size. Adeno-associated viral (AAV) vectors encoding Kir4.1-eGFP or td-Tomato under the control of an AS-specific promoter (gfa-ABC$_1$D) (Tong et al., 2014) were injected into lateral ventricles in neonatal mice at P2–P3, which progresses into the spinal canal. As shown (Figures 5A and 5B; Figures S6A–S6D), this strategy specifically promoted td-Tomato reporter and Kir4.1-eGFP expression in spinal cord ventral horn AS, but not neurons, microglia, or oligodendrocytes. AS Kir4.1 overexpression was sufficient to increase the size of FαMNs and SαMNs at 2 months post injection (Figures 5C–5E). MNs abutting Kir4.1-eGFP-transduced AS were significantly larger, suggesting a contact-mediated effect (Figures 5F and 5G). We conclude that AS Kir4.1 overexpression is sufficient to increase the size of both FαMNs and SαMNs.

AS Kir4.1 Regulates MN Size through the PI3K/mTOR/pS6 Pathway

The PI3K/mTOR/pS6 pathway is a known regulator of neuronal size. For example, knockout of the mTOR negative regulator *PTEN* results in increased neuro-axonal size in mice (Backman et al., 2001; Fricano et al., 2014; van Diepen et al., 2009) and in humans (Kwon et al., 2001; Marsh et al., 1999) with Lhermitte-Duclos disease. As shown (Figures 6A–6D), we found that levels of the mTOR effector phosphorylated ribosomal protein

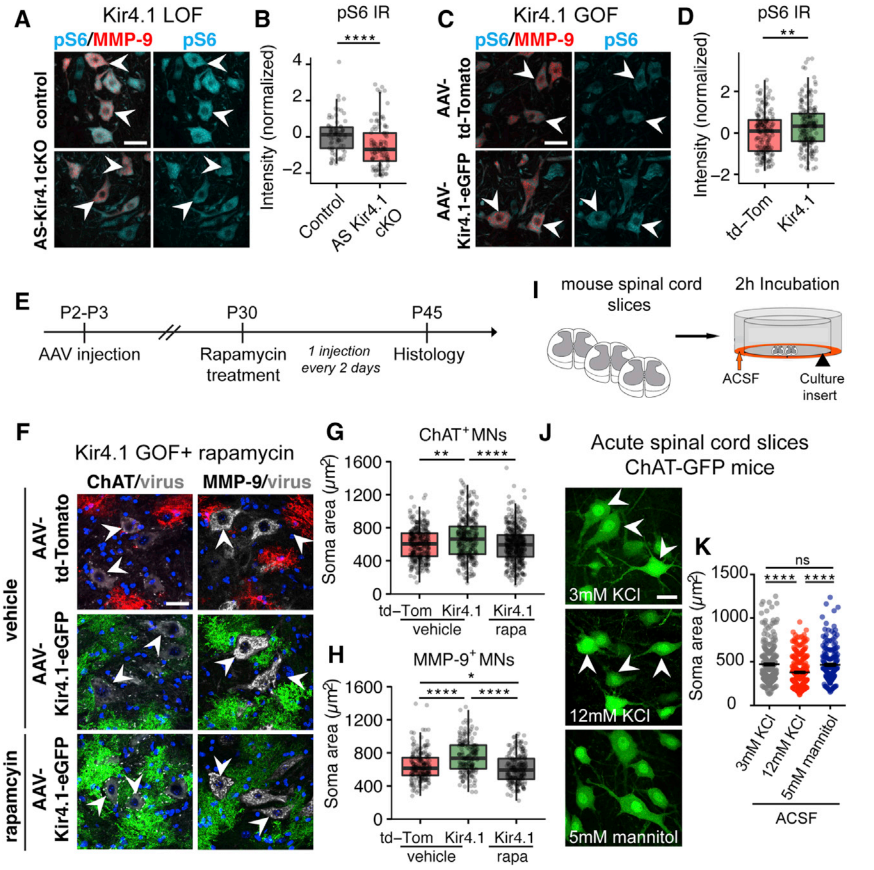

Figure 6. AS Kir4.1 Regulates MN Size through PI3K/mTOR/pS6 Pathway

(A and C) Immunofluorescence co-staining of mTOR downstream effector pS6 and MMP-9$^+$ MNs in P30 *AS-Kir4.1cKO* LOF mice (A) and in P60 AAV-Kir4.1 GOF mice (C).

(B and D) Quantification of pS6 fluorescence intensity per MMP-9$^+$ MN (B, LOF: n = 3 mice/group, 80 MNs counts/animal; D, GOF: n = 5 mice/group, 200 MN counts/animal, boxplot, Mann-Whitney test, scale bar, 25 μm).

(E) Rapamycin treatment in AAV-Kir4.1 GOF mice.

(F) Immunofluorescent co-staining of ChAT (left) and MMP-9 (right) in vehicle-treated AAV-td-Tomato and AAV-Kir4.1-eGFP mice (top two panels) or rapamycin-treated AAV-Kir4.1-eGFP mice (lower panel).

(G and H) Quantification of ChAT$^+$ (G) and MMP-9$^+$ (H) MN size in vehicle-treated AAV-td-Tomato and AAV-Kir4.1-eGFP and rapamycin-treated AAV-Kir4.1-eGFP mice (n = 3–4 mice/group, 100 MN counts/animal, boxplot, Kruskal-Wallis test, scale bar, 30 μm).

(I) Incubation of acute spinal cord slices in solutions with high K$^+$ concentration.

(J) Detection of ChAT-GFP$^+$ MNs in the ventral horn of P14 mice after 2 hr incubation in 3 mM KCl, 12 mM KCl, or 5 mM mannitol ACSF.

(K) Quantification of MN soma area in the corresponding conditions (n = 4 mice/group, average 250 MN counts/animal, mean ± SEM, Kruskal-Wallis test, scale bar, 25 μm).

S6 (pS6) were decreased in MMP-9$^+$ FαMNs in P30 *AS-Kir4.1cKO* (loss-of-function, LOF) mice and increased in P60 AAV-Kir4.1-injected (gain-of-function, GOF) mice. While pS6 levels tracked with MN soma area across groups, strong correlation was lacking at the single-cell level, suggesting that mTOR signaling was a driver rather than biomarker proxy of cell size (Figure S7; STAR Methods). To determine whether the PI3K/mTOR/pS6 pathway was necessary for the increase in MN size observed with GOF, AAV-Kir4.1-eGFP, and AAV-td-Tomato-injected mice were treated with the mTOR inhibitor rapamycin or vehicle for 15 days (Saxena et al., 2013) (Figure 6E). Indeed, rapamycin treatment prevented the AAV-Kir4.1-mediated increase in ChAT$^+$ and MMP-9$^+$ MN size that were reduced to levels observed in AAV-td-Tomato controls (Figures 6F–6H).

Previous studies have shown that Kir4.1 loss or downregulation leads to deficits in AS K$^+$ uptake (Djukic et al., 2007; Olsen et al., 2006; Seifert et al., 2009) and increased extracellular K$^+$ (Tong et al., 2014). To address whether extracellular K$^+$ might directly regulate MN soma size, we incubated acute spinal cord slices from P11–P12 WT mice in 3 mM KCl ACSF (control), 12 mM KCl ACSF (high KCl), or 5 mM mannitol (control for hyperosmotic solution) for 2 hr (Figure 6I). ChAT$^+$ MNs were smaller in the high K$^+$ condition compared with isotonic ACSF and hyperosmotic mannitol ACSF controls, suggesting a specific effect of K$^+$ in regulation of cell size (Figures 6J and 6K). These findings suggest a model where AS Kir4.1 regulates MN morphology via PI3K/mTOR/pS6 signaling (Figure 7).

DISCUSSION

Recent studies support the emerging concept that AS functional diversification is tailored to particular CNS regions to optimize local neural circuit function (Ben Haim and Rowitch, 2017; Khakh and Sofroniew, 2015; Schitine et al., 2015). Spinal MNs display a large diversity of morphological and physiological properties, providing a basis for selection of specialized AS engaged in synapse modulation (Tsai et al., 2012), MN survival (Molofsky et al., 2014), and neuronal function (Freeman and Rowitch, 2013). Here, we found that AS-encoded *Kir4.1* is regionally distributed in the ventral horn with particular localization surrounding FαMNs. Loss of AS Kir4.1 function led to a selective decrease of FαMN size and to deficits in FαMN physiological function; we observed reduced fast-twitch muscle fiber size and peak strength without affecting FαMN survival. Our findings show that it is possible to uncouple loss of peak strength from FαMN death with implications for diseases like ALS. Indeed, in other pathological conditions, AS Kir4.1 downregulation is associated with neuroinflammation (Nwaobi et al., 2016), suggesting that this is a more general maladaptive phenomenon resulting in neurological disability and might also explain why neurological function improves with resolution of neuroinflammatory lesions.

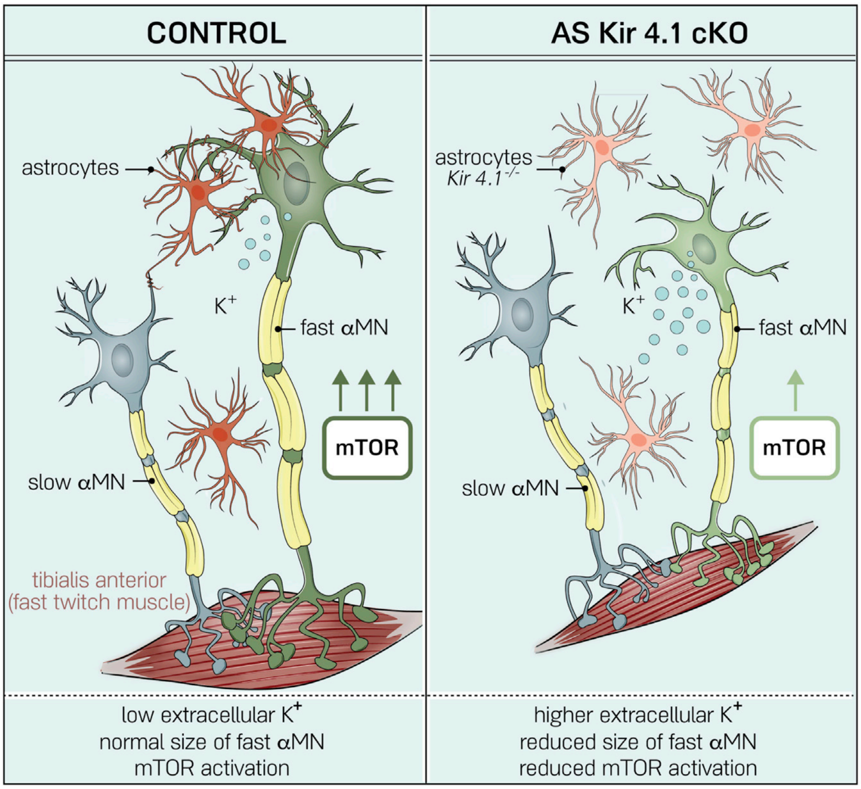

Figure 7. AS-Encoded *Kir4.1* Is Required for FαMN Function and Peak Force Generation

AS *Kir4.1* deletion leads to decreased FαMN size with mTOR downregulation, loss of fast-firing MN frequency, and decrease in fast-twitch muscle fiber size and peak strength without affecting MN survival.

Ventral Horn AS Kir4.1 Function Indicates a Subtype-Specific Relationship with FαMNs

Although the relative importance of AS inward-rectifying K^+ channels compared to AS Na^+/K^+-ATPase transporters in maintaining extracellular K^+ levels is a matter of debate (for review, see Larsen and MacAulay, 2014), it is clear that Kir4.1 is required to establish the high K^+ conductance and hyperpolarized resting membrane potential of AS (Djukic et al., 2007; Seifert et al., 2009), which are both important properties for the AS uptake of neuronal activity-released K^+. For example, *in vivo* studies have shown that Kir4.1 loss leads to altered extracellular K^+ dynamics in the hippocampus (Chever et al., 2010) and that restoration of Kir4.1 in striatal AS is sufficient to rescue extracellular K^+ increase in a transgenic mouse model of Huntington's disease (Tong et al., 2014). Previous work indicated that global or glial-restricted KO of *Kir4.1* (using the *hGFAP* promoter) results in early animal lethality by P30 with severe white matter pathology (Djukic et al., 2007; Neusch et al., 2001). However, it is difficult to determine the cell-type-specific contribution of Kir4.1 in these studies as Kir4.1 was removed from both AS and oligodendrocytes. In our work, using *Aldh1l1-cre* to target AS, we observed near total loss of Kir4.1 expression in ventral horn AS, whereas expression in oligodendrocytes was relatively preserved; thus, specific targeting of the astroglial lineage with *Aldh1l1-cre* most likely accounts for reduced severity of the phenotype in our study in which animals survived for at least 1 year of age.

Given the importance of AS-encoded inward-rectifying K^+ channel function in homeostasis, we surmised it would be required for optimal activity by all MN groups. However, we were surprised to find highly MN subtype-specific effects of *AS-Kir4.1cKO*. We found that AS-encoded *Kir4.1* was specifically needed for the function of fast, but not slow, αMNs and that its loss did not appear to affect γMNs. While numbers of α- and γMNs were normal and survival over time of all FαMNs, SαMNs, and γMNs populations was not altered, we observed a selective impact of *Kir4.1* loss of function on FαMNs. *AS-Kir4.1cKO* resulted in biophysical changes indicative of FαMN dysfunction, a conclusion further supported by loss of fast-twitch muscle mass and peak strength. Viral-mediated overexpression of Kir4.1 in spinal AS led to a non-selective increase in the size of both FαMNs and SαMNs, with a more prominent effect in MNs directly abutting transduced AS. The fact that increased AS Kir4.1 is sufficient to promote and maintain both slow and fast αMN size suggests that AS Kir4.1 levels are optimized to specific neuronal subpopulations and is consistent with our observation of selective Kir4.1 upregulation surrounding FαMNs.

Ventral Horn AS-Encoded *Kir4.1* Regulates FαMN Size through PI3K/mTOR/pS6 Signaling

The PI3K/mTOR/pS6 signaling pathway regulates metabolism and cell size (Backman et al., 2001; Fricano et al., 2014; van Diepen et al., 2009). Our data indicate that Kir4.1-driven increases in αMN size are PI3K/mTOR/pS6 pathway dependent. First, changes in the mTOR downstream effector pS6 parallel changes in MN size in both GOF and Kir4.1 LOF models. Second, GOF studies showed that Kir4.1-driven increase in cell size was reversed by the mTOR inhibitor rapamycin *in vivo*.

Might Kir4.1 have other effects in AS relevant to MN size and activity regulation? We took a comprehensive approach to this question by performing an RNA-seq profiling of FACS-purified AS from *AS-Kir4.1cKO* animals and control mice. These studies showed that, apart from *Kir4.1* levels itself, *Kir4.1cKO* AS maintain other differentiated characteristics *in vivo*. In addition, we found no evidence for astrogliosis or microglial activation at any time points analyzed in *AS-Kir4.1cKO* mice. Prior studies have shown an association between Kir4.1 and glutamate uptake (Djukic et al., 2007; Kucheryavykh et al., 2007); however, three lines of evidence indicate that deficiency in glutamate uptake is unlikely to explain the MN phenotype observed in *AS-Kir4.1cKO* mice.

First, we found no evidence for dysregulated glutamate uptake in spinal cord synaptosomes from *AS-Kir4.1cKO* animals—a method validated in GLT-1$^{-/-}$ animals (Tanaka et al., 1997)—which probably relates to the fact that GLT-1 (*Slc1a2*) levels and transporter currents are approximately 10-fold lower in the spinal cord compared to hippocampus (Regan et al., 2007). Second, RNA-seq profiling of FACS-isolated AS from *AS-Kir4.1cKO* and control mice showed no difference in expression levels of glutamate transporters GLT-1 (*Slc1a2*) and Glast (*Slc1a3*). Third, *AS-Kir4.1cKO* animals survived up to 1 year of age without MN degeneration in contrast to GLT-1$^{-/-}$ animals (Rothstein et al., 1996; Tanaka et al., 1997). Thus, AS Kir4.1 function does not result in prominent secondary changes in AS gene expression and measured glutamate uptake function. Our findings indicate region-specific AS Kir4.1 functions that interact specifically with FαMN populations. Such regionally adapted AS functions might comprise a general principle underlying their diversity.

Both Intrinsic and Extrinsic Cues Regulate AS Kir4.1 Expression in Development and Disease

The extent to which AS diversity is developmentally specified through pattern formation and/or induced locally by neuronal cues is unclear (Ben Haim and Rowitch, 2017). For example, a recent study showed that Purkinje neuron-derived Sonic hedgehog regulates AS cell fate and Kir4.1 expression in cerebellum (Farmer et al., 2016). Indeed, we found that developmental upregulation of ventral horn Kir4.1 was dependent on VGLUT1, suggesting that region-specific maturation of Kir4.1 is promoted by neuronal activity or associated factors. There might be a selective effect on Kir4.1 regulation by VGLUT1 synaptic terminal activity onto ventral horn MNs since VGLUT1 and Kir4.1 protein levels did not perfectly correlate in other parts of the spinal cord (Figure S2A) and brain regions, such as the cortex, that are known to have VGLUT1 activity but display lower levels of Kir4.1. However, in contrast to regulation by neuron-derived cues, AS diversification also obeys developmental patterning *in vivo* (Hochstim et al., 2008; Tsai et al., 2012) and *in vitro* (Krencik et al., 2011).

As recent studies have demonstrated that increased extracellular K^+ decreases mTOR signaling in T cells (Eil et al., 2016) and that loss of AS Kir4.1 leads to increased extracellular K^+ (Tong et al., 2014), we investigated whether extracellular K^+ could directly regulate MN cell size. In acute spinal cord slices, we found that increased extracellular K^+ was sufficient to reduce MN size, which suggests that AS Kir4.1 might regulate MN mTOR signaling and size through extracellular K^+. In addition to K^+, additional factors and mechanisms might be at play in Kir4.1-mediated MN size regulation. Indeed, Kir4.1 expression is selectively enriched in AS processes contacting MN soma, and MN size increase in Kir4.1 GOF mice was prominently in MNs directly abutting transduced astrocytes, suggesting that Kir4.1 effects could be partly contact mediated. Biophysical modeling studies have shown that AS Kir4.1 is particularly important for K^+ clearance during high levels of neuronal activity (Sibille et al., 2015), which corresponds to the FαMN electrophysiological signature. These MNs also have a larger VGLUT1 synaptic density than SαMNs (Basaldella et al., 2015). Hence, there might be an AS-MN subtype-specific crosstalk in the ventral spinal cord, where FαMN high activity/high VGLUT1 innervation drives AS Kir4.1 expression, which in turn decreases extracellular K^+ and modulates intrinsic MN mTOR signaling to control MN size.

Moreover, our study provides evidence that Kir4.1 is regulated in AS in a cell-autonomous manner. First, *in vitro* cultures with purified AS derived from neonatal spinal cord maintained ventral enrichment of *Kir4.1* levels in the absence of neurons. Second, in cultured human iPSC-derived AS, we found that mutant SOD1 exerted cell-autonomous effects to downregulate KIR4.1. These findings suggest that interplay between intrinsic and extrinsic factors is needed to achieve optimal Kir4.1 expression during development. We speculate that patterning results in early relative upregulation of Kir4.1 in ventral AS domains and perhaps primes AS to detect neuronal activity-dependent cues that determine physiological Kir4.1 levels critical to maintain FαMN function and peak strength. It follows that maladaptive downregulation of Kir4.1 in disease would disrupt this support mechanism with functional compromise of FαMNs.

AS-Encoded *Kir4.1* Uncouples Loss of Peak Strength from FαMN Death

MN cell death is an irreversible step of clinical decline in ALS. FαMNs have been shown to be vulnerable in SOD1G93A mice (Kaplan et al., 2014; Pun et al., 2006; Saxena et al., 2009). For example, loss of function of the FαMN-specific marker MMP-9 delays disease progression while its overexpression in FαMNs accelerated TA denervation (Kaplan et al., 2014). Several studies indicate that mouse model and ALS patient-derived MNs have physiological alterations including intrinsic hyperexcitability (Devlin et al., 2015; Wainger et al., 2014), hypoexcitability (Sareen et al., 2013), or some combination depending on maturational state (Devlin et al., 2015) with still some considerable debate about what occurs *in vivo* (Sances et al., 2016). In general, altered MN excitability is thought to contribute to chronic MN dysfunction as a common pathogenic feature in ALS.

By focusing on roles of AS, our study helps clarify the consequences of alterations in MN excitability and the importance of AS-encoded factors in regulating the clinical phenotype of peak strength in the following ways. First, while conditional KO of *Kir4.1* in AS leads to an early dysregulation of MN excitability, we failed to observe any signs of MN loss through at least 6 months of age. This analysis contained counts of total MN numbers including FαMNs, SαMNs, and γMNs. These findings make the points that (1) alterations in MN excitability in ALS could be linked to impaired K^+ buffering by AS that downregulate Kir4.1 channels and (2) excitability dysregulation in *AS-Kir4.1cKO* animals is insufficient to cause MN cell death. Indeed, crossing *AS-Kir4.1cKO* into the SOD1G93A mutant background failed to accelerate MN loss in this animal model, suggesting that Kir4.1 is not essential for MN survival even in the setting of ALS. Second, loss of peak strength can be accounted for by AS-encoded Kir4.1 downregulation. Kir4.1 expression is reduced in both mouse and rat SOD1G93A models *in vivo* (Bataveljić et al., 2012; Kaiser et al., 2006), and in this study, we found that iPSC-derived AS from ALS patients show significant loss of *Kir4.1* expression *in vitro*. Furthermore,

as stated above, loss of Kir4.1 in AS causes a decrease of peak strength in the absence of MN cell death. Together, these findings suggest that loss of AS Kir4.1 in ALS is maladaptive and might underlie the initial clinical presentation of weakness. It follows that loss of peak strength early in the ALS disease course might represent pathology caused by Kir4.1 down-regulation in AS. If so, this potentially “reversible state” could be treatable by gene overexpression to promote Kir4.1 expression in ventral horn AS to assuage muscle weakness. Together, these findings suggest the consequences of AS Kir4.1 loss on MN function in ALS and neuroinflammatory conditions.

STAR★METHODS

Detailed methods are provided in the online version of this paper and include the following:

- KEY RESOURCES TABLE
- CONTACT FOR REAGENT AND RESOURCE SHARING
- EXPERIMENTAL MODEL AND SUBJECT DETAILS
  - Human Spinal Cord Tissue
  - Mice
  - Human iPSC Lines
- METHOD DETAILS
  - Viral Vectors, Injections, and Pharmacological Treatment
  - Immunohistochemistry
  - Immunocytochemistry
  - Muscle Histology and Analysis
  - Motor Pool Labeling
  - Flow Cytometry
  - Astrocyte Cell Culture
  - Human iPSC Culture and AS Differentiation
  - RNA Sequencing and Analysis
  - qPCR Analysis
  - Western Blot
  - Glutamate Uptake Assay
  - Whole-Cell Patch-Clamp Recordings
  - High KCl Acute Spinal Cord Slice Experiment
  - Behavioral Analysis
  - Regional mRNA Bioinformatics Analysis
- QUANTIFICATION AND STATISTICAL ANALYSIS
  - Motor Neuron Soma Size and Count Analysis
  - Astrocyte and Synaptic Puncta Quantification
  - Statistical Analysis
- DATA AND SOFTWARE AVAILABILITY

SUPPLEMENTAL INFORMATION

Supplemental Information includes seven figures and two tables and can be found with this article online at https://doi.org/10.1016/j.neuron.2018.03.010.

ACKNOWLEDGMENTS

We thank Ken McCarthy (University of North Carolina) for *Kir4.1*$^{fl/fl}$ mice and genomic sequence information. We are grateful to Khalida Sabeur (UCSF) for cryopreservation and re-derivation of mouse lines and to Allan Basbaum and Lily Jan (UCSF) for helpful discussions and comments. We thank Richard Reynolds and Djordje Gveric for providing human spinal cord samples from the UK Multiple Sclerosis Tissue Bank, funded by the Multiple Sclerosis Society of Great Britain and Northern Ireland (registered charity 207495). We are also grateful to Prof. Su-Chun Zhang for providing the SOD1 mutant/isogenic control hiPSC lines. Library preparation and QCs for RNA sequencing were conducted by Yanxia Hao, Jim McGuire, and Natasha Carli at the Gladstone Genomics Core. We also acknowledge support from the James B. Pendleton Charitable Trust for NextSeq 500 runs at the Gladstone Genomics Core. Behavioral data were obtained with the help of the Neurobehavioral Core for Rehabilitation Research. K.W.K. was supported by the Medical Scientist Training Program (T32GM007618) and a California Institute of Regenerative Medicine pre-doctoral fellowship (TG201153). L.S. was supported by a post-doctoral fellowship from the German Research Foundation (DFG, SCHI 1330/1-1). A.V.M. was supported by a Pew Scholars Award and the NIMH (K08MH104417). R.P. is a Wellcome Trust Clinician Scientist (101149/Z/13/A), and A.L. is an MRC Clinician Scientist (MR/P008658/1). The study was supported by the Paul Allen Foundation Distinguished Investigator Program (E.M.U. and D.H.R.) and the ALS Association (to E.M.U. and D.H.R.) and was made possible by grants from the NEI (EY002162 to E.M.U.), NINDS (NS040511 to D.H.R.), Howard Hughes Medical Institute, Cambridge NIHR Biomedical Research Center, and the Wellcome Trust (to D.H.R.).

AUTHOR CONTRIBUTIONS

Conceptualization, K.W.K., L.B.H., and D.H.R.; Methodology, K.W.K., L.B.H., G.E.T., R.P., and A.L.; Investigation, K.W.K., L.B.H., L.S., G.E.T., R.P., A.L., M.T., J.G.M., H.-H.T., and S.M.C.; Writing – Original Draft, K.W.K. and D.H.R.; Writing – Review & Editing, K.W.K., L.B.H., and D.H.R.; Funding Acquisition, E.M.U. and D.H.R.; Supervision, A.V.M., Y.Y., R.P., A.L., E.M.U., and D.H.R.

DECLARATION OF INTERESTS

L.S. filed a patent for the detection of antibodies against KIR4.1 in a subpopulation of patients with multiple sclerosis (WO2015166057A1).

Received: March 15, 2017
Revised: November 8, 2017
Accepted: March 5, 2018
Published: March 29, 2018

REFERENCES

Aken, B.L., Ayling, S., Barrell, D., Clarke, L., Curwen, V., Fairley, S., Fernandez Banet, J., Billis, K., García Girón, C., Hourlier, T., et al. (2016). The Ensembl gene annotation system. Database (Oxford) *2016*. baw093. Published online June 23, 2016. https://doi.org/10.1093/database/baw093.

Allen, N.J. (2014). Astrocyte regulation of synaptic behavior. Annu. Rev. Cell Dev. Biol. *30*, 439–463.

Allen, N.J., and Barres, B.A. (2009). Neuroscience: Glia - more than just brain glue. Nature *457*, 675–677.

Anders, S., Pyl, P.T., and Huber, W. (2015). HTSeq—a Python framework to work with high-throughput sequencing data. Bioinformatics *31*, 166–169.

Andrews, S. (2010). FastQC: a quality control tool for high throughput sequence data (Babraham Institute).

Backman, S.A., Stambolic, V., Suzuki, A., Haight, J., Elia, A., Pretorius, J., Tsao, M.S., Shannon, P., Bolon, B., Ivy, G.O., and Mak, T.W. (2001). Deletion of Pten in mouse brain causes seizures, ataxia and defects in soma size resembling Lhermitte-Duclos disease. Nat. Genet. *29*, 396–403.

Basaldella, E., Takeoka, A., Sigrist, M., and Arber, S. (2015). Multisensory signaling shapes vestibulo-motor circuit specificity. Cell *163*, 301–312.

Bataveljić, D., Nikolić, L., Milosević, M., Todorović, N., and Andjus, P.R. (2012). Changes in the astrocytic aquaporin-4 and inwardly rectifying potassium channel expression in the brain of the amyotrophic lateral sclerosis SOD1(G93A) rat model. Glia *60*, 1991–2003.

Ben Haim, L., and Rowitch, D.H. (2017). Functional diversity of astrocytes in neural circuit regulation. Nat. Rev. Neurosci. *18*, 31–41.

Boillée, S., Yamanaka, K., Lobsiger, C.S., Copeland, N.G., Jenkins, N.A., Kassiotis, G., Kollias, G., and Cleveland, D.W. (2006). Onset and progression in inherited ALS determined by motor neurons and microglia. Science *312*, 1389–1392.

Bolstad, B.M., Irizarry, R.A., Astrand, M., and Speed, T.P. (2003). A comparison of normalization methods for high density oligonucleotide array data based on variance and bias. Bioinformatics *19*, 185–193.

Burke, R.E., Levine, D.N., and Zajac, F.E., 3rd (1971). Mammalian motor units: physiological-histochemical correlation in three types in cat gastrocnemius. Science *174*, 709–712.

Cahoy, J.D., Emery, B., Kaushal, A., Foo, L.C., Zamanian, J.L., Christopherson, K.S., Xing, Y., Lubischer, J.L., Krieg, P.A., Krupenko, S.A., et al. (2008). A transcriptome database for astrocytes, neurons, and oligodendrocytes: a new resource for understanding brain development and function. J. Neurosci. *28*, 264–278.

Chen, H., Qian, K., Du, Z., Cao, J., Petersen, A., Liu, H., Blackbourn, L.W., 4th, Huang, C.L., Errigo, A., Yin, Y., et al. (2014). Modeling ALS with iPSCs reveals that mutant SOD1 misregulates neurofilament balance in motor neurons. Cell Stem Cell *14*, 796–809.

Cheung, G., Sibille, J., Zapata, J., and Rouach, N. (2015). Activity-dependent plasticity of astroglial potassium and glutamate clearance. Neural Plast. *2015*, 109106.

Chever, O., Djukic, B., McCarthy, K.D., and Amzica, F. (2010). Implication of Kir4.1 channel in excess potassium clearance: an in vivo study on anesthetized glial-conditional Kir4.1 knock-out mice. J. Neurosci. *30*, 15769–15777.

Cleveland, D.W., and Rothstein, J.D. (2001). From Charcot to Lou Gehrig: deciphering selective motor neuron death in ALS. Nat. Rev. Neurosci. *2*, 806–819.

Devlin, A.C., Burr, K., Borooah, S., Foster, J.D., Cleary, E.M., Geti, I., Vallier, L., Shaw, C.E., Chandran, S., and Miles, G.B. (2015). Human iPSC-derived motoneurons harbouring TARDBP or C9ORF72 ALS mutations are dysfunctional despite maintaining viability. Nat. Commun. *6*, 5999.

Djukic, B., Casper, K.B., Philpot, B.D., Chin, L.S., and McCarthy, K.D. (2007). Conditional knock-out of Kir4.1 leads to glial membrane depolarization, inhibition of potassium and glutamate uptake, and enhanced short-term synaptic potentiation. J. Neurosci. *27*, 11354–11365.

Doyle, J.P., Dougherty, J.D., Heiman, M., Schmidt, E.F., Stevens, T.R., Ma, G., Bupp, S., Shrestha, P., Shah, R.D., Doughty, M.L., et al. (2008). Application of a translational profiling approach for the comparative analysis of CNS cell types. Cell *135*, 749–762.

Eccles, J.C., Eccles, R.M., and Lundberg, A. (1957). Durations of after-hyperpolarization of motoneurones supplying fast and slow muscles. Nature *179*, 866–868.

Eil, R., Vodnala, S.K., Clever, D., Klebanoff, C.A., Sukumar, M., Pan, J.H., Palmer, D.C., Gros, A., Yamamoto, T.N., Patel, S.J., et al. (2016). Ionic immune suppression within the tumour microenvironment limits T cell effector function. Nature *537*, 539–543.

Farmer, W.T., Abrahamsson, T., Chierzi, S., Lui, C., Zaelzer, C., Jones, E.V., Bally, B.P., Chen, G.G., Théroux, J.F., Peng, J., et al. (2016). Neurons diversify astrocytes in the adult brain through sonic hedgehog signaling. Science *351*, 849–854.

Freeman, M.R., and Rowitch, D.H. (2013). Evolving concepts of gliogenesis: a look way back and ahead to the next 25 years. Neuron *80*, 613–623.

Fremeau, R.T., Jr., Kam, K., Qureshi, T., Johnson, J., Copenhagen, D.R., Storm-Mathisen, J., Chaudhry, F.A., Nicoll, R.A., and Edwards, R.H. (2004). Vesicular glutamate transporters 1 and 2 target to functionally distinct synaptic release sites. Science *304*, 1815–1819.

Fricano, C.J., Despenza, T., Jr., Frazel, P.W., Li, M., O'Malley, A.J., Westbrook, G.L., and Luikart, B.W. (2014). Fatty acids increase neuronal hypertrophy of Pten knockdown neurons. Front. Mol. Neurosci. *7*, 30.

Gong, S., Zheng, C., Doughty, M.L., Losos, K., Didkovsky, N., Schambra, U.B., Nowak, N.J., Joyner, A., Leblanc, G., Hatten, M.E., and Heintz, N. (2003). A gene expression atlas of the central nervous system based on bacterial artificial chromosomes. Nature *425*, 917–925.

GTEx Consortium (2015). Human genomics. The Genotype-Tissue Expression (GTEx) pilot analysis: multitissue gene regulation in humans. Science *348*, 648–660.

Hadzipasic, M., Tahvildari, B., Nagy, M., Bian, M., Horwich, A.L., and McCormick, D.A. (2014). Selective degeneration of a physiological subtype of spinal motor neuron in mice with SOD1-linked ALS. Proc. Natl. Acad. Sci. USA *111*, 16883–16888.

Hall, C.E., Yao, Z., Choi, M., Tyzack, G.E., Serio, A., Luisier, R., Harley, J., Preza, E., Arber, C., Crisp, S.J., et al. (2017). Progressive motor neuron pathology and the role of astrocytes in a human stem cell model of VCP-related ALS. Cell Rep. *19*, 1739–1749.

Henneman, E. (1957). Relation between size of neurons and their susceptibility to discharge. Science *126*, 1345–1347.

Hochstim, C., Deneen, B., Lukaszewicz, A., Zhou, Q., and Anderson, D.J. (2008). Identification of positionally distinct astrocyte subtypes whose identities are specified by a homeodomain code. Cell *133*, 510–522.

Kaiser, M., Maletzki, I., Hülsmann, S., Holtmann, B., Schulz-Schaeffer, W., Kirchhoff, F., Bähr, M., and Neusch, C. (2006). Progressive loss of a glial potassium channel (KCNJ10) in the spinal cord of the SOD1 (G93A) transgenic mouse model of amyotrophic lateral sclerosis. J. Neurochem. *99*, 900–912.

Kang, S.H., Li, Y., Fukaya, M., Lorenzini, I., Cleveland, D.W., Ostrow, L.W., Rothstein, J.D., and Bergles, D.E. (2013). Degeneration and impaired regeneration of gray matter oligodendrocytes in amyotrophic lateral sclerosis. Nat. Neurosci. *16*, 571–579.

Kanning, K.C., Kaplan, A., and Henderson, C.E. (2010). Motor neuron diversity in development and disease. Annu. Rev. Neurosci. *33*, 409–440.

Kaplan, A., Spiller, K.J., Towne, C., Kanning, K.C., Choe, G.T., Geber, A., Akay, T., Aebischer, P., and Henderson, C.E. (2014). Neuronal matrix metalloproteinase-9 is a determinant of selective neurodegeneration. Neuron *81*, 333–348.

Kasukawa, T., Masumoto, K.H., Nikaido, I., Nagano, M., Uno, K.D., Tsujino, K., Hanashima, C., Shigeyoshi, Y., and Ueda, H.R. (2011). Quantitative expression profile of distinct functional regions in the adult mouse brain. PLoS ONE *6*, e23228.

Kenney, A.M., and Rowitch, D.H. (2000). Sonic hedgehog promotes G(1) cyclin expression and sustained cell cycle progression in mammalian neuronal precursors. Mol. Cell. Biol. *20*, 9055–9067.

Kernell, D. (1966). Input resistance, electrical excitability, and size of ventral horn cells in cat spinal cord. Science *152*, 1637–1640.

Khakh, B.S., and Sofroniew, M.V. (2015). Diversity of astrocyte functions and phenotypes in neural circuits. Nat. Neurosci. *18*, 942–952.

Kim, D., Pertea, G., Trapnell, C., Pimentel, H., Kelley, R., and Salzberg, S.L. (2013). TopHat2: accurate alignment of transcriptomes in the presence of insertions, deletions and gene fusions. Genome Biol. *14*, R36.

Krencik, R., Weick, J.P., Liu, Y., Zhang, Z.J., and Zhang, S.C. (2011). Specification of transplantable astroglial subtypes from human pluripotent stem cells. Nat. Biotechnol. *29*, 528–534.

Kucheryavykh, Y.V., Kucheryavykh, L.Y., Nichols, C.G., Maldonado, H.M., Baksi, K., Reichenbach, A., Skatchkov, S.N., and Eaton, M.J. (2007). Downregulation of Kir4.1 inward rectifying potassium channel subunits by RNAi impairs potassium transfer and glutamate uptake by cultured cortical astrocytes. Glia *55*, 274–281.

Kwon, C.H., Zhu, X., Zhang, J., Knoop, L.L., Tharp, R., Smeyne, R.J., Eberhart, C.G., Burger, P.C., and Baker, S.J. (2001). Pten regulates neuronal soma size: a mouse model of Lhermitte-Duclos disease. Nat. Genet. *29*, 404–411.

Langmead, B., and Salzberg, S.L. (2012). Fast gapped-read alignment with Bowtie 2. Nat. Methods *9*, 357–359.

Larsen, B.R., and MacAulay, N. (2014). Kir4.1-mediated spatial buffering of K(+): experimental challenges in determination of its temporal and quantitative contribution to K(+) clearance in the brain. Channels (Austin) *8*, 544–550.

Li, H., Handsaker, B., Wysoker, A., Fennell, T., Ruan, J., Homer, N., Marth, G., Abecasis, G., and Durbin, R.; 1000 Genome Project Data Processing Subgroup (2009). The Sequence Alignment/Map format and SAMtools. Bioinformatics *25*, 2078–2079.

Lobsiger, C.S., and Cleveland, D.W. (2007). Glial cells as intrinsic components of non-cell-autonomous neurodegenerative disease. Nat. Neurosci. *10*, 1355–1360.

Love, M.I., Huber, W., and Anders, S. (2014). Moderated estimation of fold change and dispersion for RNA-seq data with DESeq2. Genome Biol. *15*, 550.

Marsh, D.J., Kum, J.B., Lunetta, K.L., Bennett, M.J., Gorlin, R.J., Ahmed, S.F., Bodurtha, J., Crowe, C., Curtis, M.A., Dasouki, M., et al. (1999). PTEN mutation spectrum and genotype-phenotype correlations in Bannayan-Riley-Ruvalcaba syndrome suggest a single entity with Cowden syndrome. Hum. Mol. Genet. *8*, 1461–1472.

Matyash, V., and Kettenmann, H. (2010). Heterogeneity in astrocyte morphology and physiology. Brain Res. Brain Res. Rev. *63*, 2–10.

Mitra, P., and Brownstone, R.M. (2012). An in vitro spinal cord slice preparation for recording from lumbar motoneurons of the adult mouse. J. Neurophysiol. *107*, 728–741.

Molofsky, A.V., Krencik, R., Ullian, E.M., Tsai, H.H., Deneen, B., Richardson, W.D., Barres, B.A., and Rowitch, D.H. (2012). Astrocytes and disease: a neurodevelopmental perspective. Genes Dev. *26*, 891–907.

Molofsky, A.V., Glasgow, S.M., Chaboub, L.S., Tsai, H.H., Murnen, A.T., Kelley, K.W., Fancy, S.P., Yuen, T.J., Madireddy, L., Baranzini, S., et al. (2013). Expression profiling of Aldh1l1-precursors in the developing spinal cord reveals glial lineage-specific genes and direct Sox9-Nfe2l1 interactions. Glia *61*, 1518–1532.

Molofsky, A.V., Kelley, K.W., Tsai, H.H., Redmond, S.A., Chang, S.M., Madireddy, L., Chan, J.R., Baranzini, S.E., Ullian, E.M., and Rowitch, D.H. (2014). Astrocyte-encoded positional cues maintain sensorimotor circuit integrity. Nature *509*, 189–194.

Morel, L., Higashimori, H., Tolman, M., and Yang, Y. (2014). VGluT1+ neuronal glutamatergic signaling regulates postnatal developmental maturation of cortical protoplasmic astroglia. J. Neurosci. *34*, 10950–10962.

Müller, D., Cherukuri, P., Henningfeld, K., Poh, C.H., Wittler, L., Grote, P., Schlüter, O., Schmidt, J., Laborda, J., Bauer, S.R., et al. (2014). Dlk1 promotes a fast motor neuron biophysical signature required for peak force execution. Science *343*, 1264–1266.

Muroyama, Y., Fujiwara, Y., Orkin, S.H., and Rowitch, D.H. (2005). Specification of astrocytes by bHLH protein SCL in a restricted region of the neural tube. Nature *438*, 360–363.

Muthukumar, A.K., Stork, T., and Freeman, M.R. (2014). Activity-dependent regulation of astrocyte GAT levels during synaptogenesis. Nat. Neurosci. *17*, 1340–1350.

Neusch, C., Rozengurt, N., Jacobs, R.E., Lester, H.A., and Kofuji, P. (2001). Kir4.1 potassium channel subunit is crucial for oligodendrocyte development and in vivo myelination. J. Neurosci. *21*, 5429–5438.

Nwaobi, S.E., Cuddapah, V.A., Patterson, K.C., Randolph, A.C., and Olsen, M.L. (2016). The role of glial-specific Kir4.1 in normal and pathological states of the CNS. Acta Neuropathol. *132*, 1–21.

Oberheim, N.A., Goldman, S.A., and Nedergaard, M. (2012). Heterogeneity of astrocytic form and function. Methods Mol. Biol. *814*, 23–45.

Oldham, M.C., Langfelder, P., and Horvath, S. (2012). Network methods for describing sample relationships in genomic datasets: application to Huntington's disease. BMC Syst. Biol. *6*, 63.

Olsen, M.L., Higashimori, H., Campbell, S.L., Hablitz, J.J., and Sontheimer, H. (2006). Functional expression of Kir4.1 channels in spinal cord astrocytes. Glia *53*, 516–528.

Olsen, M.L., Campbell, S.L., and Sontheimer, H. (2007). Differential distribution of Kir4.1 in spinal cord astrocytes suggests regional differences in K+ homeostasis. J. Neurophysiol. *98*, 786–793.

Olsen, M.L., Campbell, S.C., McFerrin, M.B., Floyd, C.L., and Sontheimer, H. (2010). Spinal cord injury causes a wide-spread, persistent loss of Kir4.1 and glutamate transporter 1: benefit of 17 beta-oestradiol treatment. Brain *133*, 1013–1025.

Philips, T., and Rothstein, J.D. (2015). Rodent models of amyotrophic lateral sclerosis. Curr. Protocols Pharmacol. *69*, 1–21, 21.

Pun, S., Santos, A.F., Saxena, S., Xu, L., and Caroni, P. (2006). Selective vulnerability and pruning of phasic motoneuron axons in motoneuron disease alleviated by CNTF. Nat. Neurosci. *9*, 408–419.

Regan, M.R., Huang, Y.H., Kim, Y.S., Dykes-Hoberg, M.I., Jin, L., Watkins, A.M., Bergles, D.E., and Rothstein, J.D. (2007). Variations in promoter activity reveal a differential expression and physiology of glutamate transporters by glia in the developing and mature CNS. J. Neurosci *27*, 6607–6619.

Robberecht, W., and Philips, T. (2013). The changing scene of amyotrophic lateral sclerosis. Nat. Rev. Neurosci. *14*, 248–264.

Robinson, M.B., Hunter-Ensor, M., and Sinor, J. (1991). Pharmacologically distinct sodium-dependent L-[3H]glutamate transport processes in rat brain. Brain Res. *544*, 196–202.

Rosen, D.R. (1993). Mutations in Cu/Zn superoxide dismutase gene are associated with familial amyotrophic lateral sclerosis. Nature *364*, 362.

Rothstein, J.D., Dykes-Hoberg, M., Pardo, C.A., Bristol, L.A., Jin, L., Kuncl, R.W., Kanai, Y., Hediger, M.A., Wang, Y., Schielke, J.P., et al. (1996). Knockout of glutamate transporters reveals a major role for astroglial transport in excitotoxicity and clearance of glutamate. Neuron *16*, 675–686.

Sances, S., Bruijn, L.I., Chandran, S., Eggan, K., Ho, R., Klim, J.R., Livesey, M.R., Lowry, E., Macklis, J.D., Rushton, D., et al. (2016). Modeling ALS with motor neurons derived from human induced pluripotent stem cells. Nat. Neurosci. *19*, 542–553.

Sareen, D., O'Rourke, J.G., Meera, P., Muhammad, A.K., Grant, S., Simpkinson, M., Bell, S., Carmona, S., Ornelas, L., Sahabian, A., et al. (2013). Targeting RNA foci in iPSC-derived motor neurons from ALS patients with a C9ORF72 repeat expansion. Sci. Transl. Med. *5*, 208ra149.

Saxena, S., and Caroni, P. (2011). Selective neuronal vulnerability in neurodegenerative diseases: from stressor thresholds to degeneration. Neuron *71*, 35–48.

Saxena, S., Cabuy, E., and Caroni, P. (2009). A role for motoneuron subtype-selective ER stress in disease manifestations of FALS mice. Nat. Neurosci. *12*, 627–636.

Saxena, S., Roselli, F., Singh, K., Leptien, K., Julien, J.P., Gros-Louis, F., and Caroni, P. (2013). Neuroprotection through excitability and mTOR required in ALS motoneurons to delay disease and extend survival. Neuron *80*, 80–96.

Schirmer, L., Srivastava, R., Kalluri, S.R., Böttinger, S., Herwerth, M., Carassiti, D., Srivastava, B., Gempt, J., Schlegel, J., Kuhlmann, T., et al. (2014). Differential loss of KIR4.1 immunoreactivity in multiple sclerosis lesions. Ann. Neurol. *75*, 810–828.

Schitine, C., Nogaroli, L., Costa, M.R., and Hedin-Pereira, C. (2015). Astrocyte heterogeneity in the brain: from development to disease. Front. Cell. Neurosci. *9*, 76.

Seifert, G., Hüttmann, K., Binder, D.K., Hartmann, C., Wyczynski, A., Neusch, C., and Steinhäuser, C. (2009). Analysis of astroglial K+ channel expression in the developing hippocampus reveals a predominant role of the Kir4.1 subunit. J. Neurosci. *29*, 7474–7488.

Sibille, J., Dao Duc, K., Holcman, D., and Rouach, N. (2015). The neuroglial potassium cycle during neurotransmission: role of Kir4.1 channels. PLoS Comput. Biol. *11*, e1004137.

Sposito, T., Preza, E., Mahoney, C.J., Setó-Salvia, N., Ryan, N.S., Morris, H.R., Arber, C., Devine, M.J., Houlden, H., Warner, T.T., et al. (2015). Developmental regulation of tau splicing is disrupted in stem cell-derived neurons from frontotemporal dementia patients with the 10 + 16 splice-site mutation in MAPT. Hum. Mol. Genet. *24*, 5260–5269.

Stifani, N. (2014). Motor neurons and the generation of spinal motor neuron diversity. Front. Cell. Neurosci. *8*, 293.

Tallini, Y.N., Shui, B., Greene, K.S., Deng, K.Y., Doran, R., Fisher, P.J., Zipfel, W., and Kotlikoff, M.I. (2006). BAC transgenic mice express enhanced green

fluorescent protein in central and peripheral cholinergic neurons. Physiol. Genomics *27*, 391–397.

Tanaka, K., Watase, K., Manabe, T., Yamada, K., Watanabe, M., Takahashi, K., Iwama, H., Nishikawa, T., Ichihara, N., Kikuchi, T., et al. (1997). Epilepsy and exacerbation of brain injury in mice lacking the glutamate transporter GLT-1. Science *276*, 1699–1702.

Tien, A.C., Tsai, H.H., Molofsky, A.V., McMahon, M., Foo, L.C., Kaul, A., Dougherty, J.D., Heintz, N., Gutmann, D.H., Barres, B.A., and Rowitch, D.H. (2012). Regulated temporal-spatial astrocyte precursor cell proliferation involves BRAF signalling in mammalian spinal cord. Development *139*, 2477–2487.

Tong, X., Ao, Y., Faas, G.C., Nwaobi, S.E., Xu, J., Haustein, M.D., Anderson, M.A., Mody, I., Olsen, M.L., Sofroniew, M.V., and Khakh, B.S. (2014). Astrocyte Kir4.1 ion channel deficits contribute to neuronal dysfunction in Huntington's disease model mice. Nat. Neurosci. *17*, 694–703.

Trapnell, C., Williams, B.A., Pertea, G., Mortazavi, A., Kwan, G., van Baren, M.J., Salzberg, S.L., Wold, B.J., and Pachter, L. (2010). Transcript assembly and quantification by RNA-seq reveals unannotated transcripts and isoform switching during cell differentiation. Nat. Biotechnol. *28*, 511–515.

Tsai, H.H., Li, H., Fuentealba, L.C., Molofsky, A.V., Taveira-Marques, R., Zhuang, H., Tenney, A., Murnen, A.T., Fancy, S.P., Merkle, F., et al. (2012). Regional astrocyte allocation regulates CNS synaptogenesis and repair. Science *337*, 358–362.

Tyzack, G.E., Hall, C.E., Sibley, C.R., Cymes, T., Forostyak, S., Carlino, G., Meyer, I.F., Schiavo, G., Zhang, S.C., Gibbons, G.M., et al. (2017). A neuroprotective astrocyte state is induced by neuronal signal EphB1 but fails in ALS models. Nat. Commun. *8*, 1164.

van Diepen, M.T., Parsons, M., Downes, C.P., Leslie, N.R., Hindges, R., and Eickholt, B.J. (2009). MyosinV controls PTEN function and neuronal cell size. Nat. Cell Biol. *11*, 1191–1196.

Vinsant, S., Mansfield, C., Jimenez-Moreno, R., Del Gaizo Moore, V., Yoshikawa, M., Hampton, T.G., Prevette, D., Caress, J., Oppenheim, R.W., and Milligan, C. (2013). Characterization of early pathogenesis in the SOD1(G93A) mouse model of ALS: part II, results and discussion. Brain Behav. *3*, 431–457.

Wainger, B.J., Kiskinis, E., Mellin, C., Wiskow, O., Han, S.S., Sandoe, J., Perez, N.P., Williams, L.A., Lee, S., Boulting, G., et al. (2014). Intrinsic membrane hyperexcitability of amyotrophic lateral sclerosis patient-derived motor neurons. Cell Rep. *7*, 1–11.

Yamanaka, K., Chun, S.J., Boillee, S., Fujimori-Tonou, N., Yamashita, H., Gutmann, D.H., Takahashi, R., Misawa, H., and Cleveland, D.W. (2008). Astrocytes as determinants of disease progression in inherited amyotrophic lateral sclerosis. Nat. Neurosci. *11*, 251–253.

Yang, Y., Gozen, O., Watkins, A., Lorenzini, I., Lepore, A., Gao, Y., Vidensky, S., Brennan, J., Poulsen, D., Won Park, J., et al. (2009). Presynaptic regulation of astroglial excitatory neurotransmitter transporter GLT1. Neuron *61*, 880–894.

Yang, Y., Vidensky, S., Jin, L., Jie, C., Lorenzini, I., Frankl, M., and Rothstein, J.D. (2011). Molecular comparison of GLT1+ and ALDH1L1+ astrocytes in vivo in astroglial reporter mice. Glia *59*, 200–207.

Zhang, Y., Chen, K., Sloan, S.A., Bennett, M.L., Scholze, A.R., O'Keeffe, S., Phatnani, H.P., Guarnieri, P., Caneda, C., Ruderisch, N., et al. (2014). An RNA-sequencing transcriptome and splicing database of glia, neurons, and vascular cells of the cerebral cortex. J. Neurosci. *34*, 11929–11947.

Zurolo, E., de Groot, M., Iyer, A., Anink, J., van Vliet, E.A., Heimans, J.J., Reijneveld, J.C., Gorter, J.A., and Aronica, E. (2012). Regulation of Kir4.1 expression in astrocytes and astrocytic tumors: a role for interleukin-1 β. J. Neuroinflammation *9*, 280.

STAR★METHODS

KEY RESOURCES TABLE

REAGENT or RESOURCE	SOURCE	IDENTIFIER
**Antibodies**		
Goat polyclonal anti-ChAT	Millipore	Cat# AB144P, RRID: AB_2079751
Rabbit polyclonal anti-DsRed	Clontech Laboratories	Cat# 632496, RRID: AB_10013483
Rat Monoclonal anti-GFAP Clone 2.2B10	Innovative Research	Cat# 13-0300, RRID: AB_86543
Chicken polyclonal anti-GFP	Aves labs	Cat# GFP-1020, RRID: AB_10000240
Rabbit polyclonal anti-Kir4.1 intracellular	Alomone	Cat# APC-035, RRID: AB_2040120
Rabbit polyclonal anti-Kir4.1 extracellular	Alomone	Cat# APC-165, RRID: AB_2341043
Rabbit anti-laminin	Sigma	Cat# L9393, RRID: AB_477163
Goat anti-MMP-9	Sigma	Cat# M9570, RRID: AB_1079397
Mouse Monoclonal anti-Myosin type 1 Clone NOQ7.5.4D	Sigma	Cat# M8421, RRID: AB_477248
Mouse Monoclonal anti-Myosin Clone MY-32	Sigma	Cat# M4276, RRID: AB_477190
Mouse Monoclonal anti-NeuN	Millipore	Cat# MAB377, RRID: AB_2298772
Mouse Monoclonal anti-Neurofilament H non phosphorylated (SMI32)	BioLegend	Cat# 801701, RRID: AB_2564642
Guinea pig polyclonal anti-VGLUT1	Millipore	Cat# AB5905, RRID: AB_2301751
Guinea pig polyclonal anti-VGLUT2	Millipore	Cat# AB2251-I, RRID: AB_2665454
Mouse monoclonal anti-GFAP clone GA5	Sigma	Cat# G3893, RRID: AB_477010
Rabbit polyclonal anti-ALDH1L1	Abcam	Cat# ab87117, RRID: AB_10712968
Mouse Monoclonal anti-β-actin Clone AC-15	Sigma	Cat# A5441, RRID: AB_476744
Mouse Monoclonal anti-β-actin Clone AC-74	Sigma	Cat# A5316, RRID: AB_476743
Alexa donkey anti-goat 555	Invitrogen	Cat# A-21432, RRID: AB_2535853
Alexa donkey anti-goat 647	Invitrogen	Cat# A-21447, RRID: AB_2535864
Alexa donkey anti-goat 488	Invitrogen	Cat# A-11055, RRID: AB_2534102
Alexa donkey anti-rabbit 555	Invitrogen	Cat# A-31572, RRID: AB_162543
Alexa donkey anti-rabbit 647	Invitrogen	Cat# A-31573, RRID: AB_2536183
Alexa donkey anti-rabbit 488	Invitrogen	Cat# A-21206, RRID: AB_2535792
Alexa donkey anti-mouse 555	Invitrogen	Cat# A-31570, RRID: AB_2536180
Alexa donkey anti-mouse 647	Invitrogen	Cat# A-31571, RRID: AB_162542
Alexa donkey anti-mouse 488	Invitrogen	Cat# A-21202, RRID: AB_141607
Alexa goat anti-rabbit 555	Invitrogen	Cat# A32732, RRID: AB_2633281
Alexa goat anti-mouse 647	Invitrogen	Cat# A32728, RRID: AB_2633277
IRDye Goat anti-mouse 800	LI-COR	Cat # 925-32210
IRDye Goat anti-rabbit 680	LI-COR	Cat # 925-68071
**Bacterial and Virus Strains**		
Viral vector: AAV2/9-gfa$_{ABC1D}$-Td-Tomato	Tong et al., 2014; Penn Vector Core	Cat# V5495R
Viral vector: AAV2/9-gfa$_{ABC1D}$-Kir4.1–eGFP	Tong et al., 2014; Penn Vector Core	Cat# V5460R
**Biological Samples**		
Human spinal cord blocks	UK Multiple Sclerosis Tissue Bank	N/A
**Chemicals, Peptides, and Recombinant Proteins**		
Paraformaldehyde	Sigma	Cat# 158127
DL-*threo*-β-Benzyloxyaspartic acid (TBOA)	Tocris	Cat# 1223
L-^{3}H glutamate	PerkinElmer	NET490250UC

(*Continued on next page*)

Continued

REAGENT or RESOURCE	SOURCE	IDENTIFIER
Rapamycin	LC labs	Cat# R-5000
Cholera Toxin Subunit B-Alexa Fluor 594 Conjugate	Thermo Fisher Scientific	Cat# C22842
Papain	Worthington	Cat# LS003119
L-cysteine	Sigma	Cat# 168149
Ovomucoid trypsin inhibitor	Worthington	Cat# LS003087
DNase I	Sigma	Cat# DN25
LightCycler 480 SYBR Green I Master mix	LifeScience	Cat# 04707516001
SuperScript III First-Strand Synthesis System	Invitrogen	Cat# 18080051
Protease/Phosphatase Inhibitor Cocktail	Cell Signaling Technology	Cat# 5872
Critical Commercial Assays		
RNeasy Mini kit	QIAGEN	Cat# 74104
NextSeq 500/550 High Output v2 kit (75 cycles)	Illumina	Cat# FC-404-2005
Deposited Data		
Raw and normalized RNA-seq data	This paper	GEO: GSE111148
Experimental Models: Cell Lines		
Human: iPSC line from Fibroblast *Control #1*	Coriell Institute	Coriell ND41866*C
Human: iPSC line from Fibroblast *Control #2*	Sposito et al., 2015	PMC4550814
Human: iPSC line from Fibroblast *Control #3 isogenic SOD1D90D*	Chen et al., 2014	Su-Chun Zhang's lab
Human: iPSC line from Fibroblast *SOD1 #1 SOD1D90A* mutation	Chen et al., 2014	Su-Chun Zhang's lab
Human: iPSC line from Fibroblast *SOD1 #2 SOD1D90A* mutation	Sposito et al., 2015	Coriell ND35664
Experimental Models: Organisms/Strains		
Mouse: *Aldh1l1*-cre	Tien et al., 2012	N/A
Mouse: *Aldh1l1-GFP*	GENSAT project; Gong et al., 2003	RRID: MMRRC_011015-UCD
Mouse: *Kir4.1* $^{fl/fl}$ *(Kcnj10* ^{tm1Kdmc}*)*	Dr. Ken McCarthy; Djukic et al., 2007	RRID: IMSR_JAX:026826
Mouse: *EAAT2-td-Tomato*	Yang et al., 2011	N/A
Mouse: *VGLUT1* KO	Fremeau et al., 2004; Dr. Robert Edward	N/A
Mouse: HB9-cre: Rosa-DTA $^{fl/fl}$	Molofsky et al., 2014	N/A
Mouse: *ChAT-GFP*	Tallini et al., 2006; Dr. Roger Nicoll	RRID: IMSR_JAX:007902
Mouse: *SOD1G93A* mice	The Jackson Laboratory	JAX stock # 002726; RRID: IMSR_JAX:002726
Mouse: *B6SJLF1/J* mice	The Jackson Laboratory	JAX stock #100012; RRID: IMSR_JAX:100012
Oligonucleotides		
Primers for *kcnj10*: 5′- GTCGGTCGCTAAGGT CTATTACA-3′; 5′ GGCCGTCTTTCGTGAGGAC-3′	This paper	N/A
Primers for *β-actin*: 5′- TGGATCGGTGGCTCCAT CCTGG-3′; 5′- GCAGCTCAGTAACAGTCCGCCTAGA-3′	This paper	N/A
Recombinant DNA		
Plasmid: gfa$_{ABC1D}$-Td-Tomato	Tong et al., 2014	Addgene plasmid #52874
Plasmid: gfa$_{ABC1D}$-Kir4.1-eGFP	Tong et al., 2014	Addgene plasmid #44332
Software and Algorithms		
ImageJ 1.49 m	ImageJ	N/A
GraphPad prism	GraphPad Software	N/A

(*Continued on next page*)

Continued

REAGENT or RESOURCE	SOURCE	IDENTIFIER
Multiclamp 700B	Molecular Devices	N/A
DigiData 1440A	Molecular Devices	N/A
pClamp 10	Molecular Devices	N/A
CatWalk 7.1 software	Noldus	N/A
MotorMonitor	Kinder-Scientific	N/A
R 3.1.1	R core team	https://www.r-project.org/
Tophat2 v.2.0.11	Kim et al., 2013	https://ccb.jhu.edu/software/tophat/index.shtml
Bowtie2 v.2.2.3	Langmead and Salzberg, 2012	http://bowtie-bio.sourceforge.net/bowtie2/index.shtml
Htseq-count v.0.6.1p1	Anders et al., 2015	http://htseq.readthedocs.io/en/master/count.html
DESeq2 v.1.14.1	Love et al., 2014	https://bioconductor.org/packages/release/bioc/html/DESeq2.html
Cufflinks v.2.2.1	Trapnell et al., 2010	http://cole-trapnell-lab.github.io/cufflinks/
Samtools v.0.1.19	Li et al., 2009	http://samtools.sourceforge.net/
Fastqc v.0.11.4	Andrews, 2010	https://www.bioinformatics.babraham.ac.uk/projects/fastqc/

CONTACT FOR REAGENT AND RESOURCE SHARING

Further information and requests for resources and reagents should be directed to the Lead Contact David H. Rowitch (dhr25@medschl.cam.ac.uk).

EXPERIMENTAL MODEL AND SUBJECT DETAILS

Human Spinal Cord Tissue

All tissue was provided by the UK Multiple Sclerosis Tissue Bank at Imperial College, London (Table S1). Tissue was obtained via a prospective donor scheme following ethical approval by the National Research Ethics Committee (08/MRE09/31).

Mice

All mouse strains were maintained at the University of California, San Francisco (UCSF) specific pathogen-free animal facility and all animal protocols were approved by and in accordance with the guidelines established by the Institutional Animal Care and Use Committee and Laboratory Animal Resource Center. *Aldh1l1-GFP* transgenic mice were generated by the GENSAT project (Gong et al., 2003). The same methods and technology were used to generate *Aldh1l1-cre* mice as previously described (Tien et al., 2012). *Kir4.1*$^{fl/fl}$ mice were obtained from Dr. Ken Mc Carthy (University of North Carolina, Chapel Hill). The EAAT2-td-Tomato transgenic mice were generated as previously described (Yang et al., 2011). The *VGLUT1* KO mice were a gift from Dr. Robert Edward (University of California, San Francisco) (Fremeau et al., 2004). ChAT-GFP (MGI: 3694555) mice were a gift from Dr. Roger Nicoll (University of California, San Francisco) (Tallini et al., 2006). SOD1G93A and B6SJL control mice (Stock No: 002726) were obtained from The Jackson Laboratory. All mice were maintained on a 12 hr light/dark cycle with food and water available *ad libitum*. Mice were gender matched for all experiments except for experiments using SOD1G93A mice, where only males where used. Mice were kept on a mixed background and littermate controls (either WT or cre-negative) were used for all experiments.

Human iPSC Lines

Human iPSCs-derived AS were obtained from either two or three healthy control lines or a patient carrying the SOD1D90A mutation (Sposito et al., 2015). These lines included an isogenic control line (SOD1D90D) and its mutant pair (generated and provided by Prof. Su-Chun Zhang; Chen et al., 2014) (Table S2).

METHOD DETAILS

Viral Vectors, Injections, and Pharmacological Treatment

Adeno-associated viral vectors targeting AS encoding td-Tomato (AAV2/9-gfaABC1D-Td-Tomato) or Kir4.1-eGFP (AAV2/9-gfaABC1D-Kir4.1–eGFP) (Tong et al., 2014) were produced from Addgene plasmids (#52874 and #44332) by the University of Pennsylvania viral core. Wild-type (B6SJL background) pups (P2-P3) received icv injections of AAV-td-Tomato or AAV-Kir4.1-eGFP. Anesthesia was induced by hypothermia (3-5 min on ice) and injections were performed using a custom-made neonate stereotactic

frame with beveled glass needles (5 μL, wiretrol, Drummond). Viral vectors were injected in the lateral ventricles (coordinates from Lambda: lateral: +0.6 mm/antero-posterior: −1.2 mm/ventral from the skull: −1.8 mm) at a total titer of 9.10^{10} genome copies in 3 μL (2 μL right hemisphere, 1 μL left hemisphere). The needle was left in place for 3 min after injection and pups were rubbed in the litter before them putting back into the home cage. For pharmacological inhibition of the mTOR pathway, AAV-Kir4.1-eGFP or AAV-td-Tomato-injected mice received intraperitoneal injections of either Rapamycin (6 mg/kg) or vehicle for 15 days (one injection every second day) as previously described (Saxena et al., 2013). Animals were randomly allocated into experimental groups.

Immunohistochemistry

Mice were either transcardially perfused with ice-cold PBS and either perfused or samples post-fixed in 4% paraformaldehyde (PFA). After post-fixation in 4% PFA, samples were cryoprotected in 30% sucrose for 48 hr at 4°C and embedded in optimal cutting temperature (OCT) compound (Tissue-Tek). Cryosections (14-16 μm) were collected on superfrost slides (VWR) using a cryostat (CM3050S, Leica). Cryosections were subjected to heat-induced antigen retrieval in 10mM sodium citrate (pH = 6) or 1X Dako Target antigen retrieval solution for 2 min at 95°C, permeabilized and blocked in 0.1M PBS/0.2% Triton X-100/10% serum for 1 hr at room temperature (RT). Primary antibody incubations were carried out overnightcat 4c°C. After washing in 0.1M PBS, cryosections were incubated with secondary antibodies diluted in 0.1MPSB/0.2% Triton X-100/10% serum for 1 hr, RT. Donkey or horse serums were used for incubations. Goat or donkey Alexa fluochrome-tagged secondary IgG antibodies were used for primary antibody detection. Slides were mounted with DAPI Fluoromount-G (SoutherBiotech). Primary antibodies used included: goat ChAT (AB144P, Millipore, 1:200), rabbit DsRed (632496, Clonetech, 1:500), rat GFAP (13-0300, Invitrogen, 1:1000), chicken GFP (GFP-1020, Aves, 1:500), rabbit Kir4.1 (APC035, Alomone, 1:2000), rabbit laminin (L9393, Sigma, 1:1000), goat MMP-9 (M9570, Sigma, 1:500), mouse Myosin type 1 (M8421, Sigma, 1:4000), mouse Myosin type 2 (M4276, Sigma, 1:400), mouse NeuN (MAB377, Millipore, 1:1000), Purified anti-Neurofilament H (NF-H), Nonphosphorylated Antibody (SMI32, 801701, Biolegend, 1:10 000), guinea pig VGLUT1 (AB5905, Millipore, 1:5000), guinea pig VGLUT2 (AB2251, Millipore, 1:5000). Images were acquired on a Leica TCS SPE laser confocal microscope with either 20x or 40x objectives; all pictures are z stack confocal images, unless stated otherwise.

Immunocytochemistry

For immunocytochemistry (ICC), hiPSC-AS were plated onto Geltrex coated round glass coverslips (diameter: 13 mm) and fixed in 4% PFA for 10 min, RT. Standard ICC protocols were followed. Briefly, samples were permeabilized and blocked in 0.1M PBS/0.3% Triton X-100/5% normal goat serum for 1 hr. Primary antibodies were applied overnight at 4°C in the same blocking solution. Primary antibodies used were rabbit Kir4.1 (Alomone, APC035, 1:2000) and mouse GFAP (Sigma, 1:500). Secondary antibody incubation was performed for 1 hr, RT, using specie-specific Alexa fluochrome-tagged secondary IgG antibodies and nuclei were counterstained with DAPI. Samples were imaged using a Leica DM5500B microscope with a 63x objective.

Muscle Histology and Analysis

Tibialis anterior (TA) muscle was isolated after transcardial perfusion of mice with ice-cold PBS and then snap frozen in OCT with liquid nitrogen. Transverse cryosections of TA were prepared and tissue was fixed on slide with 4% PFA for 10 min, RT prior to immunostaining as described above. Muscle fiber area was quantified by tracing the laminin signal in ImageJ from confocal images obtained with a 20x objective.

Motor Pool Labeling

We used intramuscular injections of Cholera Toxin Subunit B (CSTB)-Alexa Fluor 594 Conjugate (Thermofisher) for retrograde labeling of motor pools in the spinal cord. *AS-Kir4.1cKO* or control mice (P30) were injected with 20 μL (5 individual injection sites) of CTSB in the right TA muscle. Mice were perfused 5 days later and spinal cords processed for histology as described above.

Flow Cytometry

Postnatal day 5 (Figure 1) spinal cords were microdissected using an "open book" preparation to separate dorsal and ventral pieces (Molofsky et al., 2014). Whole spinal cord was dissected at P12-14 for RNA-sequencing (Figure S4D). Tissue dissociation was performed as described previously (Cahoy et al., 2008). Briefly, tissue was collected in HBSS without Ca^{2+} and Mg^{2+} and transferred to dissociation buffer (glucose 22.5 mM, EDTA 0.5 mM, phenol red), papain (20 U mL^{-1}, Worthington), L-cysteine (1 mM), DNase (125 U/mL) for 80 min at 33°C. Tissue was then washed in dissociation buffer containing ovomucoid (1.0 mg/mL), centrifuged 5 min at 200 g. Supernatant was removed, tissue resuspended in the same buffer and mechanically disrupted using a P1000 pipette. Dissociated cells were layered onto dissociation buffer with concentrated ovomucoid (5 mg/mL). Samples were centrifuged 5 min at 200 g and pellet resuspended in staining medium with DAPI. *Aldh1l1-GFP*$^+$ and *Aldh1l1-GFP*$^-$ cells were sorted as previously described (Molofsky et al., 2013) on a BD FACS Aria II and gated on forward/side scatter, live/dead by DAPI exclusion, and GFP, using GFP^- and $DAPI^-$ controls to set gates for each experiment.

Astrocyte Cell Culture

Ventral and dorsal spinal cords from P0-1 mice were isolated and dissociated as above and described previously (Molofsky et al., 2014). Cells were plated at a density of >1 × 10^6 per 25 cm^2 flask in DMEM-hi glucose with 10% FCS/10 μM hydrocortisone,

5 μg/mL^{-1} N-acetylcysteine, 2 μg/mL^{-1} insulin and 20 ng/mL^{-1} EGF. Six days after plating cells, flasks were shaken to remove oligodendrocyte contamination. At 8 days, AraC was added to kill rapidly proliferating cells. 10–12 days after initial plating, RNA was collected for *Kir4.1* qPCR analysis. Animals were randomly allocated into experimental groups.

Human iPSC Culture and AS Differentiation

Human hiPSCs were maintained on Geltrex (Life Technologies) with Essential 8 Medium media (Life Technologies) and passaged using EDTA (Life Technologies, 0.5 mM). For the *in vitro* assays and western blot three cultures of human AS were used for each group, which derived from three separate inductions of hiPSCs, using two healthy control lines and a patient carrying the SOD1D90A mutation (Sposito et al., 2015) (Table S2). For qPCR we used three independent AS cultures for each group deriving from hiPSCs of three healthy controls and two patients with the SOD1D90A mutation. These lines included an isogenic control line (SOD1D90D) and its mutant pair (Chen et al., 2014) (Table S2). Spinal AS differentiation was carried out as previously published (Hall et al., 2017; Tyzack et al., 2017).

RNA Sequencing and Analysis

Total RNA from FACS AS was extracted with Trizol (Invitrogen) and purified using the RNeasy Kit (QIAGEN). cDNA was generated from full-length RNA using the NuGEN RNA-Seq V2 kit which uses the single primer isothermal amplification method to deplete ribosomal RNA, and sheared by Covaris to yield uniform size fragments. The NuGen Ultralow kit was used to add adapters, barcoding, and amplification. Library was purified using Agencourt XP magnetic beads, quality controlled with an agilent bioanalyzer, and quantified by qPCR. Eight libraries were pooled per lane for single end (SE75) sequencing. Over 51M reads were sequenced per sample (range 38-66M reads) using a NextSeq 500.

Read quality was assessed using fastqc (version 0.11.4) (Andrews, 2010) and the first 5 bases as well as the last base of each read were trimmed and aligned to the *Mus musculus* genome (Ensembl GRCm38) (Aken et al., 2016) using TopHat2 (version 2.0.11) with bowtie2 (version 2.2.3) (Kim et al., 2013). TopHat2 was run with the following arguments ''–no-coverage-search–segment-mismatches 1'' with the genes.gtf file from Ensembl GRCm38. Gene counts were created from the alignment files using SAMtools (version 0.1.19) (Li et al., 2009) to generate BAM files and subsequently htseq-count (version 0.6.1p1) with default parameters (Anders et al., 2015) to create count files. From raw count files, DESeq2 (Love et al., 2014) was used to detect differentially expressed genes. FPKM values were generated using cuffquant and cuffnorm from the cufflinks suite of software tools (Trapnell et al., 2010).

qPCR Analysis

RNA was isolated using Trizol reagent (Invitrogen), DNase-digested to remove genomic DNA, and purified using the RNAeasy Kit (QIAGEN) according to manufacturer's instructions. Complementary DNA was generated using Superscript III (Invitrogen) and random hexamers. Primers used included *kcnj10* (forward: GTCGGTCGCTAAGGTCTATTACA; reverse: GGCCGTCTTTCGTGAGGAC) and β-actin (forward: TGGATCGGTGGCTCCATCCTGG; reverse: GCAGCTCAGTAACAGTCCGCCTAGA). qPCR was performed on a LightCycler 480 using LightCycler 480 SYBR Green I Master mix and melting curves were analyzed to ensure primer specificity.

Western Blot

Dorsal and ventral spinal cord (Figure 1E) samples were microdissected from acute slices prepared in the same manner as done for electrophysiology (see below). Sample lysis was performed in RIPA buffer (Thermofisher) in the presence of protease and phosphatase inhibitors (Cell signaling). Samples concentration was determined with the Bradford method and protein migration and gel transfer was performed as described previously (Kenney and Rowitch, 2000). After blocking in Odyssey Blocking Buffer (PBS) (Li-Cor) for 1 hr, RT, primary antibodies were incubated O/N at 4°C onto the western blot membrane. The following antibodies were used: rabbit Kir4.1 (extracellular, APC-165, Alomone, 1:2000), rabbit Kir4.1 (APC035, Alomone, 1:2000), mouse GFAP (Sigma, 1:500), rabbit ALDH1L1 (ab87117,Abcam, 1:500), mouse β-actin (Sigma, A5441, 1:5000 or AC-74, 1:20000). IRDye Goat anti-mouse and anti-rabbit (680 and 800) fluorescent secondary antibodies (Li-Cor) were used for protein detection on the Odyssey CxI imaging system.

Glutamate Uptake Assay

Glutamate uptake was performed with crude synaptosome preparation from adult (3-6 months) mouse spinal cord using 0.32M sucrose centrifugation method (Robinson et al., 1991). After total protein determination, 1μCi L-^{3}H glutamate and 100mM non-labeled glutamate were mixed with Na^+ uptake buffer (total volume 275 mL) then added into 25 mL of each synaptosome sample in 96-well multiscreen HTS filter plates (Millipore). After 6 min incubation, uptake was terminated by putting into ice bath. Samples were then filtered using the Steriflip vacuum filtration system (Millipore) and washed 6X with ice-cold PBS while continue filtering the samples. Each filtered 96-well membrane was excised out and transferred for scintillation counting. DL-*threo*-β-Benzyloxyaspartic acid (DL-TBOA, 500 mM) was added into appropriate wells in glutamate uptake assay. Disintegration per min (DPM) value was normalized by total protein concentration and converted to pmol/mg/min.

Whole-Cell Patch-Clamp Recordings

Acute fresh lumbar (L3–4) spinal cord slices were prepared from *Aldh1l1-cre:Kir4.1$^{fl/fl}$:ChAT-GFP* mice and Cre-negative littermate controls from P12–14 using previously described protocols and solutions (Mitra and Brownstone, 2012). In brief, transverse slices (350-μm thick) were cut with a vibratome (Leica Microsystems) in a chamber filled with ice-cold sucrose cutting solution (in mM: 191 sucrose, 0.75 K-gluconate, 1.25 KH_2PO_4, 26 choline bicarbonate (80% solution), 4 $MgSO_4$, 1 $CaCl_2$, 20 dextrose, 2 kynurenic acid, 1 (+)-sodium l-ascorbate, 5 ethyl pyruvate, and 3 *myo*-inositol) followed by a brief (60 s) incubation in polythethylene glycol (Mn = 1,900–2,200). The slices were then incubated in cutting solution at 35°C for 30 min followed by 30 min in artificial cerebrospinal fluid (in mM: 121 NaCl, 3 KCl, 1.25 NaH_2PO_4, 25 $NaHCO_3$, 1.1 $MgCl_2$, 2.2 $CaCl_2$, 15 dextrose, 1 (+)-sodium l-ascorbate, 5 ethyl pyruvate, and 3 *myo*-inositol) then equilibrated to room temperature. All bicarbonate-buffered solutions were bubbled with carbogen for at least 30 min prior to use and continuously throughout their usage. Whole-cell recordings were made using patch clamp amplifiers (Multiclamp 700B) under an infrared-differential interference contrast microscope. Data acquisition and analysis were performed using digitizers (DigiData 1440A) and analysis software pClamp 10 (Molecular Devices). Signals were filtered at 6 kHz and sampled at 20 kHz. Glass pipettes with a resistance of 2.5–4 MΩ were filled with a K-methanesulphonate internal solution (in mM: 131 K-methanesulfonate, 6 NaCl, 0.1 $CaCl_2$, 1.1 EGTA-KOH, 10 HEPES, 0.3 $MgCl_2$, 3 ATP-Mg^{2+} salt, 0.5 GTP-Na^+ salt, 2.5 l-glutathione reduced, and 5 phosphocreatine di(tris) salt; the solution was adjusted to a pH of 7.25 with KOH). Series resistance (15–25 MΩ) was monitored throughout the whole-cell recording and data were discarded if the change in series resistance was >20% during the course of the experiment.

Afterhyperpolarization (AHP) amplitude and decay time were calculated from the 25 pA depolarizing step protocol (the minimum step at which action potentials occurred). AHP amplitude was determined from the voltage difference between the beginning of the action potential and the minimum of the AHP. AHP half-decay time was determined as half the time from the minimum of the AHP to its resolution.

High KCl Acute Spinal Cord Slice Experiment

Acute spinal cord slices from P11-P12 mice were obtained as described in the above paragraph. After incubations in the cutting solutions and regular ACSF, slices were transferred onto culture inserts (Millipore) in 6-well plates and incubated for 2 hr at 37°C in the following solutions: control ACSF (3 mM KCl), high KCl ACSF (12 mM KCl) or 5 mM mannitol ACSF as a control for hypertonic ACSF solution (3-4 lumbar spinal cord slices were incubated per well per condition).

Behavioral Analysis

All behavioral experiments were performed at the UCSF Neurobehavioral Core for Rehabilitation Research. Peak force, gait analysis, and the open field test were done on the same cohort of adult animals, whereas the rotarod test was performed on a different cohort of P30 animals. Maximum limb force was determined by measuring mouse forelimb grip strength using a force transducer (Ametek). The grip strength of each mouse was measured four times each day, and the highest measured value was scored. The test was performed on five consecutive days, from which the average maximum force per animal was computed. The Catwalk apparatus (Noldus) consists of a 1.3 m glass plate and two side barriers, forming a straight tunnel. In a dark room, the mouse walks across the glass plate and a high-speed camera from underneath captures its footprints. The animal's home cage was placed at one end of the glass plate and the animal was placed on the opposite end, allowing it to walk freely across the runway into its home cage. A training period of two days was used to acclimatize the animals to the training apparatus and to train the animals in the task. On the third day, three complete trials were recorded per animal. A trial was regarded as complete if the animal walked across the recorded area without stopping significantly or turning around. Gait analysis and verification of each recorded trial was done manually using CatWalk 7.1 software. The open field test (Kinder-Scientific) was used to assay overall locomotor activity. The field was divided up into zones monitored by video tracking software. Data collected includes active time, distance traveled, rearing time, time along the perimeter of the field, and time spent in the center. Each mouse was tested in the open field apparatus undisturbed for 10 min. The rotarod (Ugo Basile) test was performed on a rotating rod that accelerated from 0-40 rotations per minute (rpm) in during a 5-min period in order to assess for motor deficits. The task requires the mouse to balance on the rod and the time on the rod over the session is recorded. Animals were tested 3 times per day for 3 consecutive days. All behavioral studies were carried out with the experimenter blind to genotype.

Regional mRNA Bioinformatics Analysis

For the human regional Kir4.1 mRNA analysis, RPKM expression data from GTEx were downloaded from their data portal (gtexportal.org, V6 data release). We detected a large batch effect for GTEx (GTEx-Consortium, 2015) samples due to center acquisition site using the SampleNetwork R function (Oldham et al., 2012). We therefore restricted our analysis to samples acquired by centers 'B1, A1' or 'C1, A1'. For the mouse regional *Kir4.1* mRNA analysis, raw profiling. CEL files were obtained from the Gene Expression Omnibus website from the GEO: GSE16496 (Kasukawa et al., 2011) accession ID. Expression values were generated in R using the expresso function of the affy R package with "mas" settings and no normalization. Expression values were also quantile normalized (Bolstad et al., 2003) using the SampleNetwork R function.

QUANTIFICATION AND STATISTICAL ANALYSIS

Motor Neuron Soma Size and Count Analysis

Motor neuron soma size and counts were performed on maximal intensity projections of 10 μm z stacks with a 2 μm z-step of lumbar (L3-L6) levels on 2-4 sections per animal. Soma size was determined by tracing the ChAT-GFP signal using ImageJ software. Measurements were performed on the optical section with the largest soma area and only on motor neurons that had a DAPI nucleus visible. Similarly, motor neurons were only counted if a $DAPI^+$ nucleus was present. Phopho-S6 fluorescence intensity (IR) was measured using the mean gray value function on ImageJ within a ROI delineating individual fast $MMP\text{-}9^+$ MN soma, which assures value normalization according to the size of the selected area (i.e., MN soma). In addition, as immunofluorescence intensity can vary between sets of experiments (for example, depending on tissue fixation), the absolute values of pS6 IR were normalized to the value in their respective internal controls (=1) for both LOF and GOF experiments. Quantifications were performed on 40x confocal images (12 μm z stack, 1 μm z-step) at the lumbar (L3-L6) spinal cord levels on 5-8 sections per mouse. For the contact/non-contact soma area ratio quantification, MN soma area was quantified on merged images with transduced astrocytes in both groups. A potential limitation to this analysis is the fact that contact was defined based on 16 μm-thick sections. There may still be contact from astrocytes out of the section, which could inflate the observed effect in what we are labeling as non-contacted MNs. All quantifications regarding MN number and soma area were performed under blind conditions for experimental group (genotype, viral vectors injection or treatment groups).

Astrocyte and Synaptic Puncta Quantification

For AS Kir4.1 intensity quantification in *Aldh1L1-GFP*-labeled AS (Figure S2C), we quantified signal on maximal intensity projections of 10 μm z stacks with a 1 μm z-step using cellular areas centered on *Aldh1l1-GFP*$^+$ AS somas in ImageJ, since *Aldh1l1-GFP* signal is restricted to the soma and primary processes. Synaptic area coverage of VGLUT1 and VGLUT2 synapses were similarly obtained from maximal intensity projections of 10 μm z stacks with a 1 μm z-step using the analyze particle function in ImageJ. Thresholds were set identically for each image. For AS Kir4.1 intensity measurements in EAAT2-Td-Tomato-labeled AS (Figures 1J and 1K), 3D reconstruction was performed on 10–12 μm *z* -stacks with a 0.5–1 μm z-step using the surface tool in Imaris software, as previously described (Morel et al., 2014). The volume of individual AS can be directly measured from generated 3D domains in Imaris. To quantify Kir4.1 signal within each AS domain, individual domains were masked onto the Kir4.1 channel by setting all pixel signals outside the masked cell domain to zero so that only Kir4.1 signal within the masked AS domain remained. The Kir4.1 signal for each AS domain was quantified using ImageJ software. For AAV transduction quantification, td-$Tomato^+$ or GFP^+ area was normalized to the ventral horn total area. The mean fluorescence intensity was also quantified in the ventral spinal cord for the two groups.

Statistical Analysis

We did not perform statistics to pre-determine group sample size. However, the sample sizes used were similar to previously published studies by our group and others. All biological replicates (n) are derived from at least three independent experiments (except for the *VGLUT1 KO* experiment, n = 2). Unless otherwise specified, no data were excluded from analysis. All bar graphs are expressed as mean ± SEM. The normality of the data points was verified using the Shapiro-Wilk test and for variables that displayed normal distributions an unpaired Welch's t test to compare the difference in means between two groups. Two-way ANOVA tests and Bonferroni post hoc tests were used for the rotarod experiment (Figure 6). When normality tests failed, non- parametric Mann-Whitney (two groups) or Kruskal-Wallis (more than groups) tests were used. All statistical tests were performed using the R software environment.

DATA AND SOFTWARE AVAILABILITY

The accession number for the RNA-sequencing data reported in this paper is GEO: GSE111148.

Neuron, Volume *98*

Supplemental Information

Kir4.1-Dependent Astrocyte-Fast Motor Neuron Interactions Are Required for Peak Strength

Kevin W. Kelley, Lucile Ben Haim, Lucas Schirmer, Giulia E. Tyzack, Michaela Tolman, John G. Miller, Hui-Hsin Tsai, Sandra M. Chang, Anna V. Molofsky, Yongjie Yang, Rickie Patani, Andras Lakatos, Erik M. Ullian, and David H. Rowitch

Supplemental figures

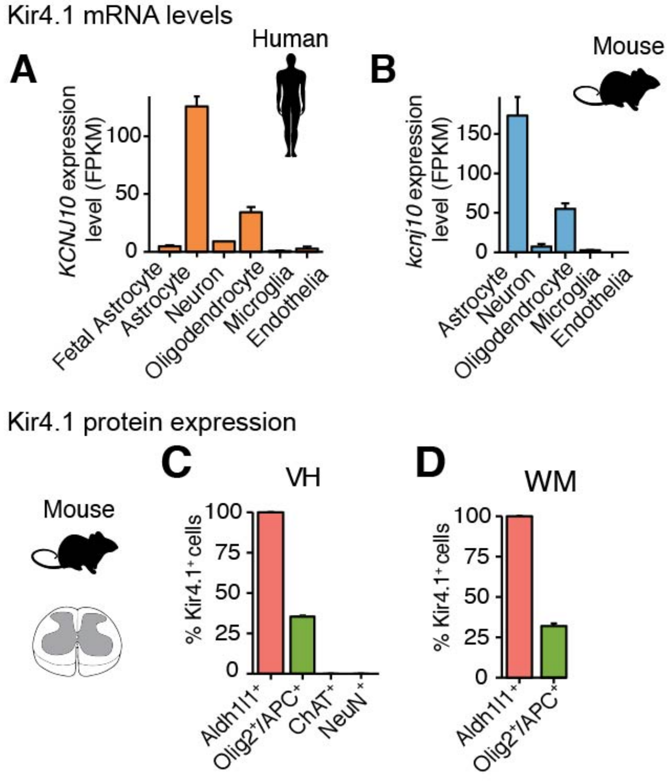

Figure S1 | *Kir4.1* is predominantly expressed in AS. Related to Figure 1. A, B) *Kir4.1* mRNA is highly expressed in cortical (CTX) AS compared to other major CNS cell types both in humans (**A**) (Zhang et al., 2016) and mice (Zhang et al., 2014) (**B**) (mean ± sem). **C, D)** Histological counts of Kir4.1 percent positive AS (Aldh1l1^{+}), oligodendrocytes (Olig2^{+}/APC (CC1)$^{+}$), neurons (NeuN^{+}) and MN (ChAT^{+}) in ventral horn (VH) (**C**) and white matter (WM) (**D**) of lumbar mouse spinal cord at P16 for AS counts and P30 for oligodendrocyte and neuron counts (n = 3 mice, mean ± sem).

Kir4.1 developmental expression pattern in relation to VGLUT1

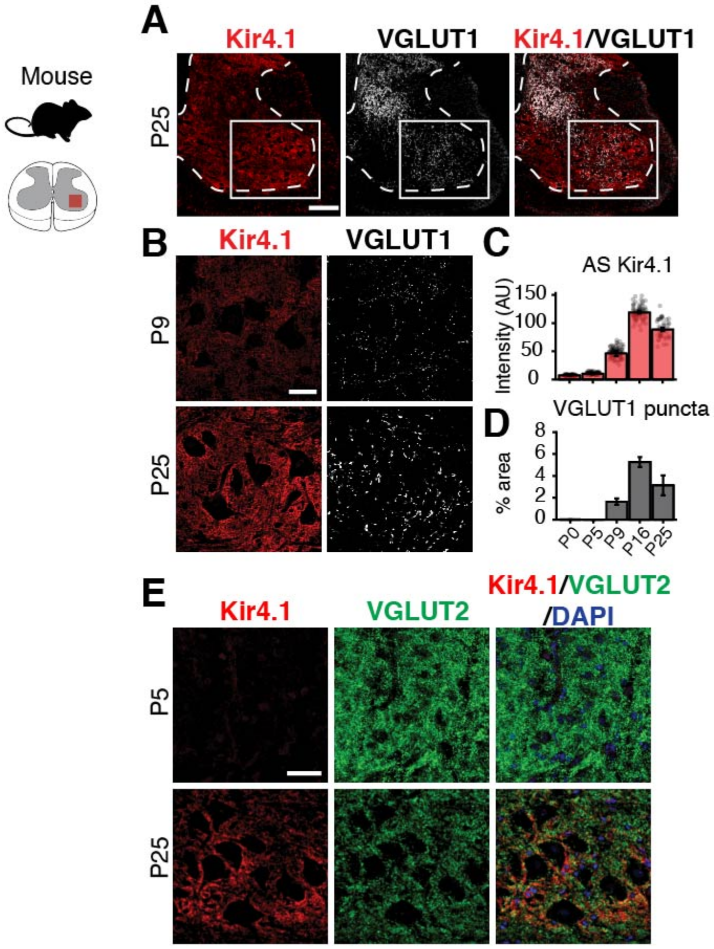

Figure S2 | Postnatal expression of AS Kir4.1 in relation to VGLUT1 and VGLUT2. Related to Figure 1. A) Immunofluorescence staining of Kir4.1 and VGLUT1 in the mouse spinal cord at P25 showing enrichment of both proteins in the ventral horn (scale bar: 200µm). **B**) Synchronized developmental upregulation of Kir4.1 and VGLUT1 in ventral lumbar spinal cord between P9 and P25 (scale bar: 40µm). **C, D)** Quantification of Kir4.1 immunofluorescence intensity per AS (*Aldh1l1-GFP*$^{+}$) (**C**) and the fraction of area occupied by VGLUT1 puncta (**D**) at the indicated developmental ages (n = 3 animals, > 50 AS counts/animal, mean ± sem). **E**) Developmental upregulation of Kir4.1 in the ventral horn does not correlate with VGLUT2 puncta expression.

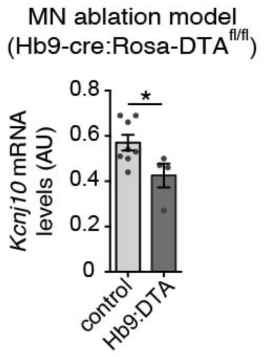

Figure S3 | MNs are necessary for *Kir4.1* expression in the ventral spinal cord. Related to Figure 1. *Kcnj10* mRNA levels are strongly downregulated in mice lacking MNs (Hb9:cre:Rosa-DTA $^{fl/fl}$) at E18.5 (n=4-8, mean ± sem, Student t-test).

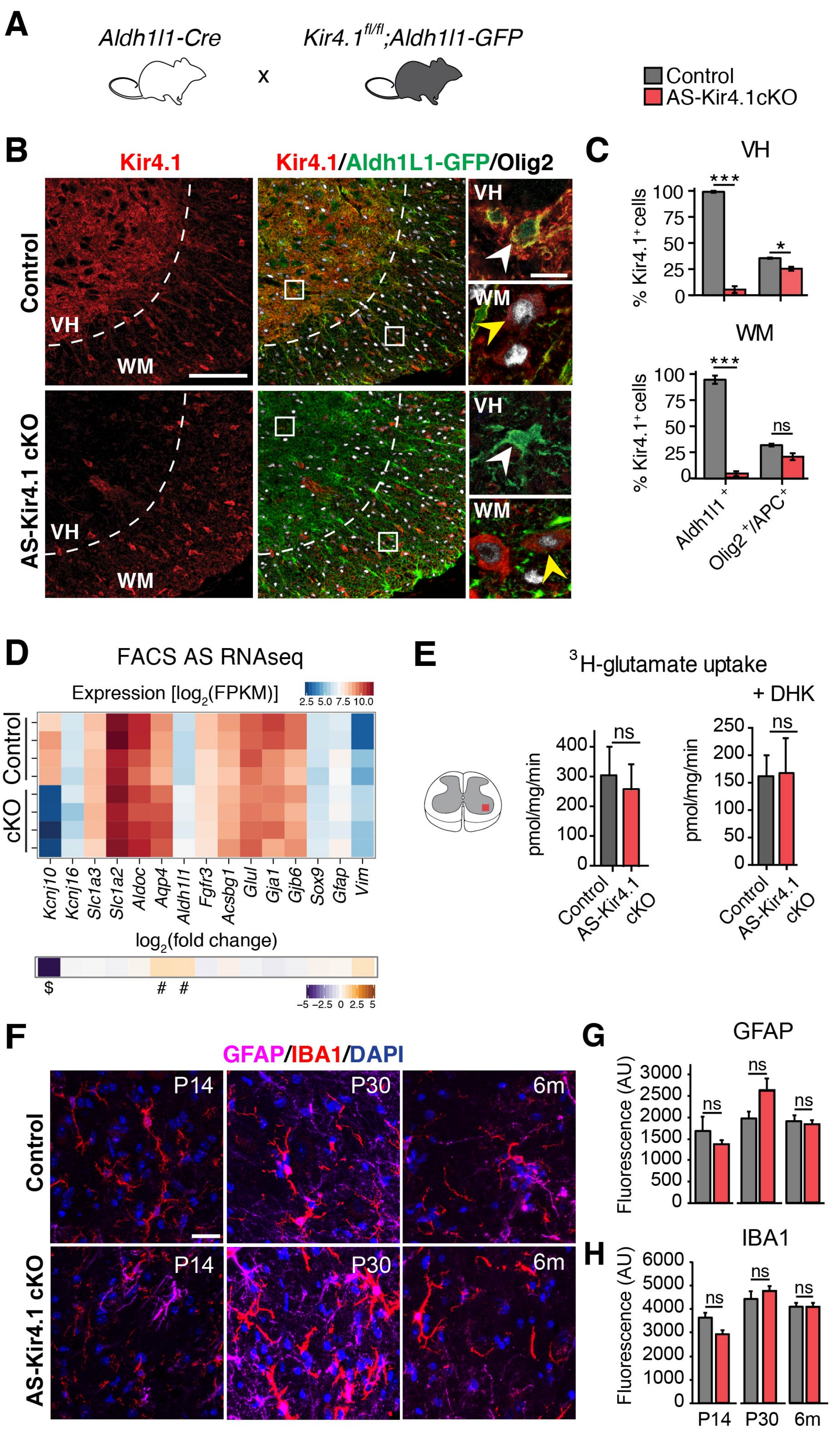
A
Aldh1l1-Cre
x
Kir4.1fl/fl;Aldh1l1-GFP
Control
AS-Kir4.1cKO
B
Kir4.1
Kir4.1/Aldh1L1-GFP/Olig2
Control
AS-Kir4.1 cKO
VH
WM
C
VH
WM
% Kir4.1+cells
***
*
ns
Aldh1l1+
Olig2+/APC+
D
FACS AS RNAseq
Expression [log2(FPKM)]
Control
cKO
Kcnj10
Kcnj16
Slc1a3
Slc1a2
Aldoc
Aqp4
Aldh1l1
Fgfr3
Acsbg1
Glul
Gja1
Gjb6
Sox9
Gfap
Vim
log2(fold change)
$
# #
E
3H-glutamate uptake
+ DHK
pmol/mg/min
ns
Control
AS-Kir4.1 cKO
F
GFAP/IBA1/DAPI
P14
P30
6m
G
GFAP
Fluorescence (AU)
H
IBA1
P14 P30 6m

Figure S4 | Characterization of *AS Kir4.1 cKO* mice. Related to Figure 2. A) Astrocyte-specific *Kir4.1* KO (*AS-Kir4.1cKO*) and cre-negative control animals were generated by crossing *Aldh1l-cre* and *Kir4.1*$^{fl/fl}$*-Aldh1l1-GFP* mice. **B**) Loss of Kir4.1 expression in *Aldh1l1-GFP*$^{+}$ AS mice but not Olig2^{+} oligodendrocytes in *AS-Kir4.1cKO* lumbar spinal cord at P30. Dotted line denotes gray/white matter boundary. VH: ventral horn. WM: white matter. Right: High-magnification AS and oligodendrocytes (scale bar: 100µm, insert: 10µm). **C)** Histological counts of Kir4.1 percent positive AS (Aldh1l1^{+}) and oligodendrocytes (Olig2^{+}/APC^{+}) in VH (top panel) and WM (bottom panel) lumbar spinal cord at P14 for AS (n = 4 mice) and P30 for oligodendrocytes (n =3 mice, mean ± sem, Welch's t-test). **D)** Top: Heat map showing mRNA expression levels of canonical AS markers in FACS-isolated AS from P14 *AS Kir4.1 cKO* and control mice. Bottom: Heat map of log2 fold changes of the indicated genes ($ and # denote significance). **p* < 0.05, *** *p* < 0.001, # $p < 10^{-6}$, $ $p < 10^{-100}$. **E)** Glutamate uptake is not altered in *AS Kir4.1 cKO* mice. Total (right) and GLT-1 mediated (left) uptake was measured using the ^{3}H-glutamate uptake assay on spinal cord samples from adult *AS-Kir4.1cKO* and cre-negative control animals (n=4-6 mice/group, mean ± sem, Mann-Whitney test). **F)** Absence of AS (GFAP) and microglia (IBA1) reactivity in the spinal cord of *AS Kir4.1 cKO* at all time points analyzed. **G, H)** Quantification of GFAP (**G**) and IBA1 (**H**) fluorescence levels in the ventral horn of *AS Kir4.1 cKO* and control animals at P14, P30 and 6m (n=3 mice/group, mean ± sem, Mann-Whitney test).

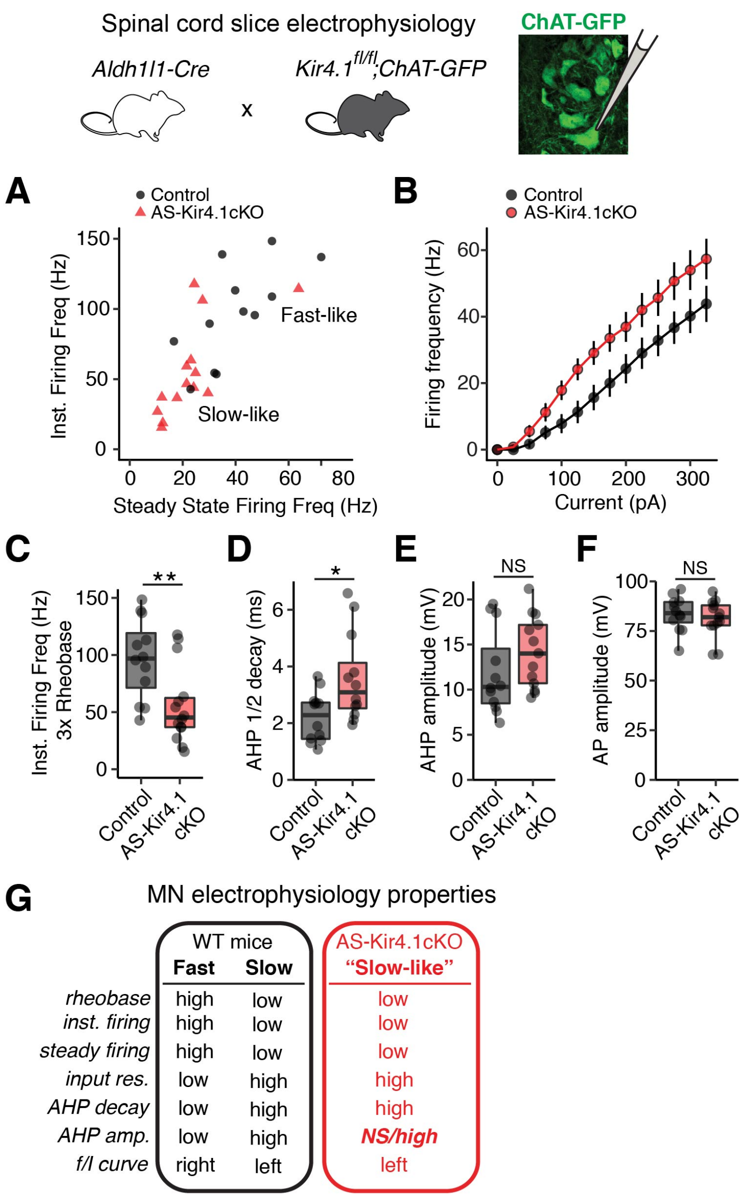

Figure S5 |AS *Kir4.1* is required for FαMN physiological properties. Related to Figure 3. (A) Scatter plot of MN instantaneous and steady state firing frequency calculated at three times rheobase. (**B**) MN firing frequency at 25 picoampere (pA) depolarizing current steps (n = 12 control MNs, n = 14 *AS-Kir4.1cKO* MNs from at least 3 animals per group, mean ± sem). (**C-F**) Instantaneous firing frequency calculated at three times rheobase (**C**), afterhyperpolarization (AHP) half decay time (**D**), AHP amplitude (**E**), and action potential (AP) amplitude (**F**) from *AS-Kir4.1cKO-ChAT-GFP* animals at P12-15 (n = 12 control MNs, n = 14 *AS-Kir4.1cKO* MNs from at least 3 animals per group, boxplot, Mann-Whitney test). Edges of boxplots denote interquartile range (25th – 75th percentile) with whiskers denoting 1.5 times the interquartile range and black line denoting the median value. * $p < 0.05$. (**G**) Table

highlighting differences in MN electrophysiological profile between fast and slow MNs identified in previous studies (left) (Hadzipasic et al., 2014; Muller et al., 2014) as compared to our findings from *AS-Kir4.1cKO* MNs (right).

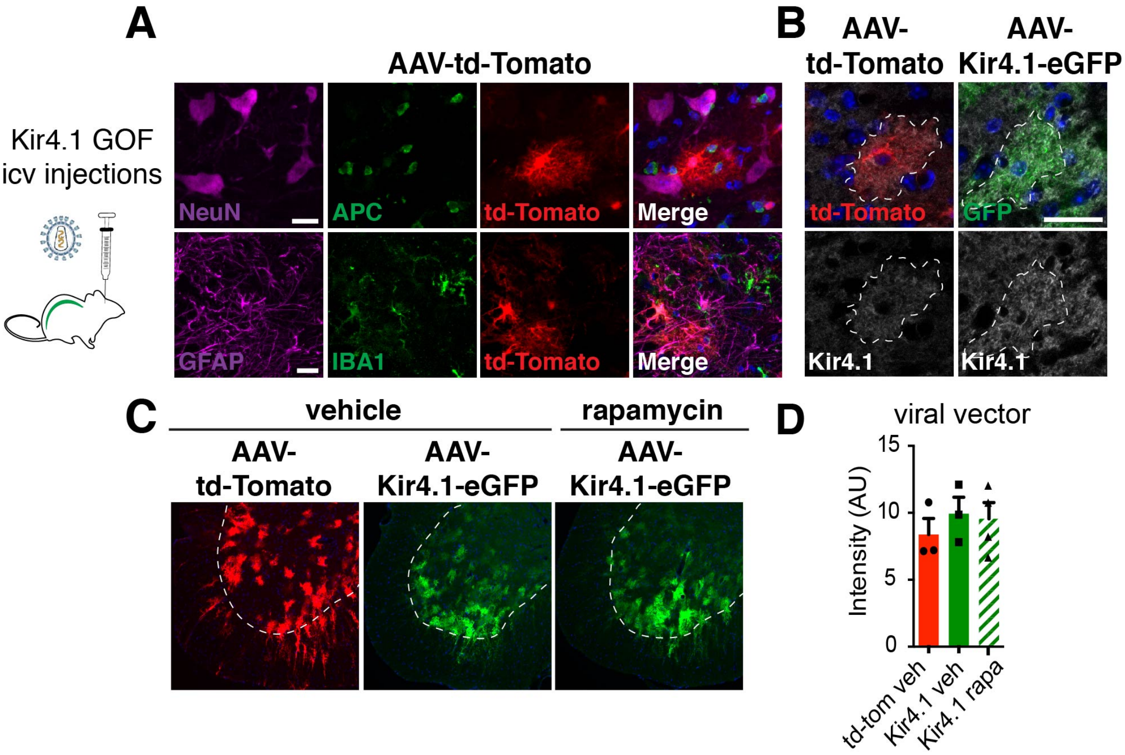

Figure S6 | Characterization of viral-mediated Kir4.1 overexpression in spinal cord AS. Related to Figure 5. A) Validation of AAV2/9-gfaABC$_1$D vector tropism in the mouse spinal cord. AAV-encoded td-Tomato is expressed in GFAP$^+$ AS but not in NeuN$^+$ neurons, IBA1$^+$ microglia or APC$^+$ oligodendrocytes in P30 mice (1 month post-injection). **B)** Increased Kir4.1 expression in AAV-Kir4.1-eGFP-injected mice as compared to AAV-td-Tomato controls. **C)** Representative images of viral transduction in the ventral horn of mice injected with AAV-td-tomato or AAV-Kir4.1-eGFP and treated with vehicle (left) or rapamycin (right). **D)** Rapamycin treatment does not alter viral transduction in the ventral horn (n=3 mice/group, mean ± sem, Mann-Whitney test).

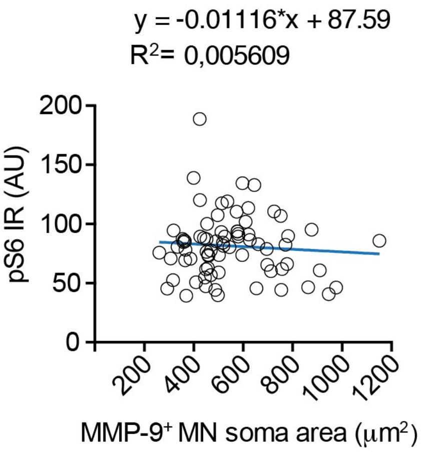

Figure S7 | Absence of correlation between pS6 fluorescence intensity and MN soma area. Related to Figure 6. pS6 IR value and corresponding soma area for individual MMP-9$^+$ MN in the spinal cord of control (cre negative) mice.

Patient #	Age (years)	Gender	Cause of death
1	86	female	Cerebrovascular ischemia
2	88	female	Bronchopneumonia
3	91	female	Cerebrovascular ischemia

Table S1. Clinical and pathological characteristics of spinal cord control patients. Related to Figure 1.

Patient #	Mutation	Age (years)	Gender	Source
Control No. 1	None	64	Male	Coriell ND41866*C
Control No. 2	None	78	Male	PMC4550814
Control No. 3 (isogenic)	None ($SOD1^{D90D}$)	50	Female	Su-Chun Zhang's lab
SOD1 No. 1	$SOD1^{D90A}$	50	Female	Su-Chun Zhang's lab
SOD1 No. 2	$SOD1^{D90A}$	70	Female	Coriell ND35664

Table S2. Human iPSC lines. Related to Figure 4.
